# Blindsight relies on a functional connection between hMT+ and the lateral geniculate nucleus, not the pulvinar

**DOI:** 10.1371/journal.pbio.2005769

**Published:** 2018-07-25

**Authors:** Sara Ajina, Holly Bridge

**Affiliations:** Wellcome Centre for Integrative Neuroimaging, FMRIB, Nuffield Department of Clinical Neurosciences, University of Oxford, Oxford, United Kingdom; McGill University, Canada

## Abstract

When the primary visual cortex (V1) is damaged, the principal visual pathway is lost, causing a loss of vision in the opposite visual field. While conscious vision is impaired, patients can still respond to certain images; this is known as ‘blindsight’. Recently, a direct anatomical connection between the lateral geniculate nucleus (LGN) and human motion area hMT+ has been implicated in blindsight. However, a functional connection between these structures has not been demonstrated. We quantified functional MRI responses to motion in 14 patients with unilateral V1 damage (with and without blindsight). Patients with blindsight showed significant activity and a preserved sensitivity to speed in motion area hMT+, which was absent in patients without blindsight. We then compared functional connectivity between motion area hMT+ and a number of structures implicated in blindsight, including the ventral pulvinar. Only patients with blindsight showed an intact functional connection with the LGN but not the other structures, supporting a specific functional role for the LGN in blindsight.

## Author summary

When the primary visual cortex (V1) is damaged in one hemisphere, we lose the ability to see one half of the world around us. Clinical tests show that in this blind region of vision, we cannot see even the brightest flashes of light. However, many years of research have shown that individuals who are blind in this way may still respond to certain images in the ‘blind’ area of vision, even though they are often unable to describe what they ‘see’ and may be unaware of seeing anything at all. This is called blindsight, and researchers are trying to understand the pathways underlying this phenomenon. A recent study mapped a physical pathway of connections in the brain that could account for blindsight in humans. However, the functional nature of this pathway has never been shown. In this study, we assess a group of patients with damage to V1, some of whom demonstrate blindsight and some of whom do not. We compare neural responses and functional connectivity and show that a functional connection in this pathway is critical for blindsight. We also reveal new insights into how speed and motion are likely to be processed in the healthy brain.

## Introduction

Damage to the primary visual cortex (V1) that may occur following a stroke causes visual loss in the corresponding part of the visual field (homonymous hemianopia, [1]). However, extensive research has shown that some patients retain an ability to respond to images inside their scotoma, even though they may not consciously see them [2]. This phenomenon is called blindsight, and recent work applied diffusion MRI and tractography in patients with V1 damage to try to uncover which pathways may underlie this residual visual function [3]. A connection between the lateral geniculate nucleus (LGN) and human motion area, hMT+, was found to be intact in patients with blindsight but was absent or impaired in patients without blindsight. The other pathways tested, which included a connection between hMT+ and the superior colliculus (SC), and with hMT+ in the opposite hemisphere, did not show this pattern. Unfortunately, a limitation of diffusion MRI is that it investigates purely structural connections, which may not relate directly to the function under investigation [4]. Furthermore, seed-based tractography is restricted to pathways chosen by investigators (see also [5–7]), which in this case did not include a connection with the thalamic pulvinar nucleus. Neither a specific role for the LGN nor a functional connection to hMT+ has been shown in human blindsight and would significantly advance our understanding of how patients respond to visual images in the absence of V1.

The current study investigated behavioural and functional MRI responses to speed of motion in a group of patients with V1 damage in adulthood (*n* = 14), and healthy age-matched controls (*n* = 8). Patients were categorised as blindsight positive or negative according to their ability to detect the visual stimulus within their blind visual field. We then compared measures of activity and functional connectivity between the two patient groups and healthy controls.

Patients with blindsight demonstrated significant fMRI activity in hMT+ in the damaged hemisphere, with a relatively preserved hMT+ response to speed in the blind hemifield. Critically, patients with blindsight also showed intact functional connectivity between hMT+ and LGN in the damaged hemisphere, which was absent in patients without blindsight. This was specific to the LGN, as both patient groups demonstrated preserved functional connectivity between hMT+ and (i) ventral pulvinar, (ii) SC, and (iii) contralateral hMT+, which was no different than in healthy controls. The pulvinar, in particular, is implicated in human and nonhuman primate studies in which V1 is damaged early in life [8,9]; however, this region is yet to be evaluated in adult-onset blindsight. Our findings support a critical functional role for the LGN and its specific connection with hMT+ in adult human blindsight, reinforced by recent evidence for an intact anatomical connection between these structures [3].

## Results

Behavioural performance for stimuli in the blind hemifield was measured using two two-alternate forced choice (2AFC) paradigms (S1 Fig). Participants viewed a central fixation cross whilst being shown an aperture of black dots on a grey background, moving at speeds of 0, 4, 8, 20, or 32°/s. Patients were categorised as blindsight positive or negative according to their ability to detect which of two intervals contained the moving dots (see ‘Materials and methods‘ for more details). According to this measure, 8 out of 14 patients with unilateral V1 damage and homonymous hemianopia were labelled as ‘blindsight positive’. Optimum performance for both tasks (i.e., most significant) was at intermediate speeds of 8 and 20°/s, and performance tended towards a quadratic relationship with speed (F = 2.6, *p* = 0.09, df = 2, S1C and S1D Fig). We performed functional MRI (fMRI) using the same visual stimuli in all 14 patients, with images presented separately to the blind or sighted hemifield in order to relate neural activity and functional connectivity to blindsight function.

### Residual responses to motion after V1 damage

**Fig 1:**
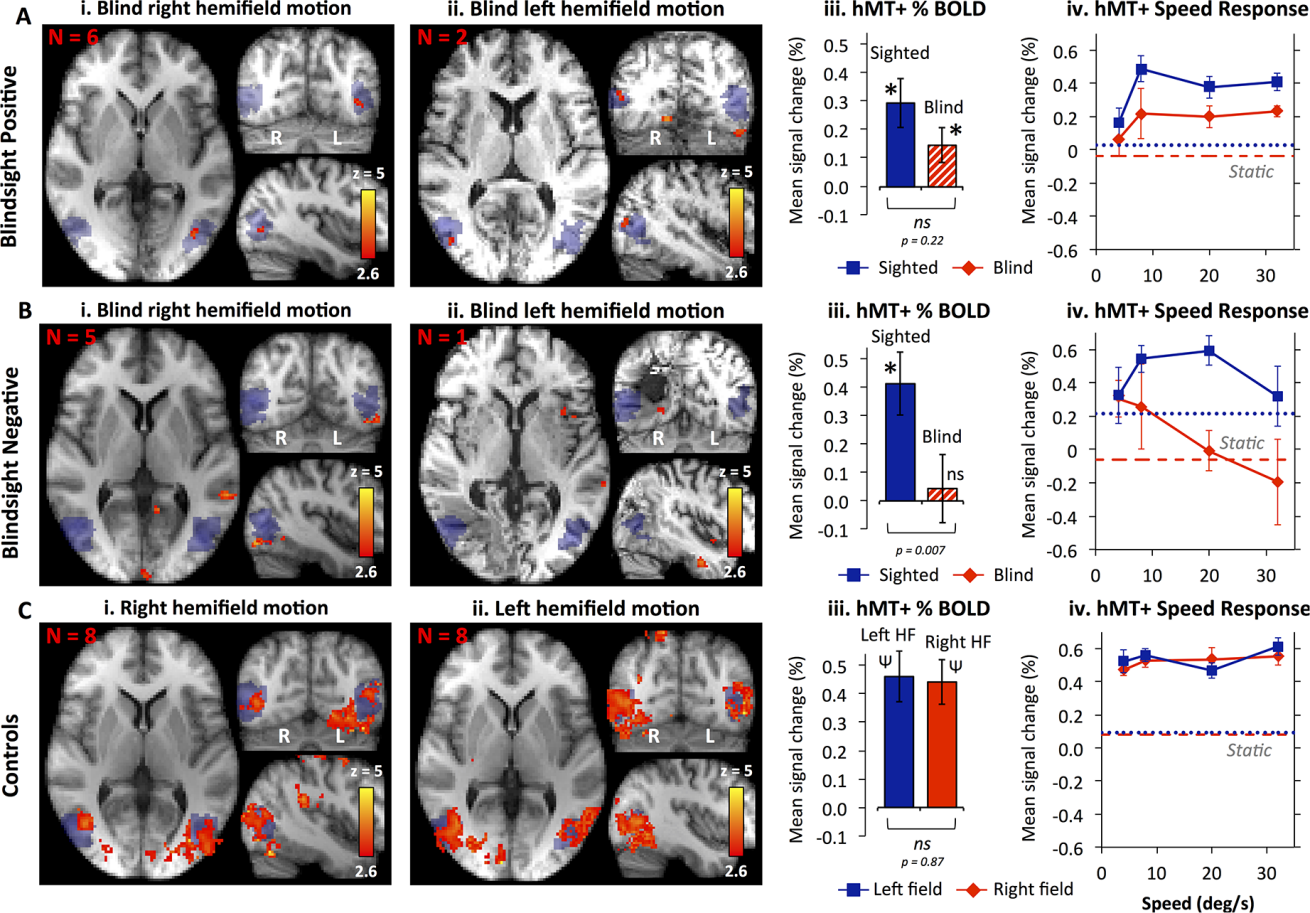
fMRI responses to motion for patients with V1 lesions and controls. Results are shown separately for **(A)** blindsight-positive patients, **(B)** blindsight-negative patients, and **(C)** healthy controls. **(i)** Significant activity for moving versus static dots in the blind right hemifield of patients with left V1 lesions and **(ii)** the blind left hemifield of patients with right V1 lesions. Mixed effects analyses, *P* < 0.001 uncorrected for a priori regions of interest, elsewhere cluster-corrected *p* < 0.01. Shaded blue areas are binarized Jülich-defined probabilistic maps of hMT+, radiological convention. **(iii)** Mean contralateral hMT+ signal change averaged across all five stimulus conditions, comparing sighted (blue) and blind (red) hemifields ± SEM. In controls, ‘Left HF’ refers to left hemifield, ‘Right HF’ is right hemifield. * significant activity above baseline *p* < 0.05, ψ *p* ≤ 0.001, ns = not significant. *P* values are from *t* tests. **(iv)** Mean signal change in contralateral hMT+ as a function of stimulus speed, shown separately for each hemifield (blue squares are sighted hemifield, red diamonds are blind hemifield). Responses to static stimuli are dotted (left/sighted hemifield) or dashed (right/blind hemifield) lines. Underlying data for iii and iv can be found in S1 Data. fMRI, functional MRI; SEM, standard error of the mean; V1, primary visual cortex.

Blindsight-positive patients demonstrated significant fMRI activity in contralateral hMT+ for moving versus static dots in the blind hemifield (Fig 1Ai and 1Aii). Overall hMT+ blood oxygen level–dependent (BOLD) signal change for all 5 speeds was significantly greater than baseline (Fig 1Aiii, *t* = 3.0, *p* = 0.02, df = 7). Activity was somewhat lower in intensity and spatial extent than the sighted field or healthy controls (Fig 1C), but there was no significant difference between blind and sighted responses in blindsight-positive patients (Fig 1Aiii *t* = 1.3, *p* = 0.2, df = 39). Whilst the absence of a significant difference may result from a lack of power (*n = 8*), blindsight-negative patients with a lower ‘n’ (Fig 1B) showed a marked effect of hemifield (paired *t* = 2.9, *p* = 0.007, df = 29) and no demonstrable hMT+ activity for moving versus static dots in the blind hemifield (Fig 1Bi and 1Bii) nor averaged across all conditions (Fig 1Biii, *t* = 0.6, *p* = 0.6, df = 5).

In addition to hMT+ activity, blindsight-positive patients with right V1 damage (Fig 1Aii, *n* = 2) showed activity in Jülich-defined right V2 and left V4, although there was no such activity in patients with left V1 damage (*n* = 6). In blindsight-negative patients, visual-evoked responses also appeared within small regions of ipsilesional V4 and V2, as well as the occipital pole and inferior parietal lobule, implying that activity in these regions was insufficient for motion perception.

It was noteworthy that overall hMT+ signal change in the undamaged hemisphere was also slightly reduced in both patient groups compared to age-matched controls. A possible explanation is that unilateral V1 damage can negatively impact sighted processing in the opposite hemisphere, perhaps via a disturbance of interhemispheric interactions [10,11].

Speed of motion (0°–32°/s) had a significant impact on hMT+ activity in the sighted hemifield of patients and equivalent left hemifield of controls (2-way ANOVA: F(4, 14) = 5.1, *p* < 0.001), with no effect of participant group (Fig 1iv, F(2, 14) = 2.2, *p* = 0.12). In the blind hemifield of patients (right hemifield of controls) there was a similar effect of speed (F(4, 14) = 3.5, *p* = 0.01) but also group (F(2, 14) = 7.4, *p* = 0.001). The pattern of responses in blindsight-positive and -negative patients differed markedly. Blindsight-positive patients showed a positive relationship between hMT+ signal change and speed (r = 0.8, 5 speeds), whilst the correlation coefficient was negative in blindsight-negative patients (r = −0.6). This difference was not simply driven by a difference between motion and static responses, as excluding the static conditions increased the significance even further (r = 0.67 versus r = −0.99, z = −2.44, *p* = 0.01). Specifically, blindsight-positive patients showed a relatively ‘normal’ hMT+ relationship with speed in the blind hemifield that was similar to the pattern in healthy controls (Pearson r = 0.89, *p* = 0.04, 5 speeds) and patients’ own sighted hemifield (r = 0.98, *p* < 0.01). This was not the case for blindsight-negative patients, either when compared to controls (r = 0.21, *p* = 0.7) or to their own sighted hemifield (r = 0.29, *p* = 0.6).

### Critical functional geniculo-extrastriate connectivity

To determine how activity in hMT+ correlated with subcortical activity, we examined the fMRI time series after stimulus-evoked responses had been regressed out. Specifically, we compared the residual pattern of activity in hMT+ with (i) LGN, (ii) ventral pulvinar, (iii) SC, and (iv) hMT+ in the opposite (undamaged) hemisphere, using subject-specific regions of interest (ROIs) (S3 Fig). We also performed whole-brain analyses to measure the voxels where neural activity most closely matched the time series of hMT+ and these subcortical structures.

**Fig 2:**
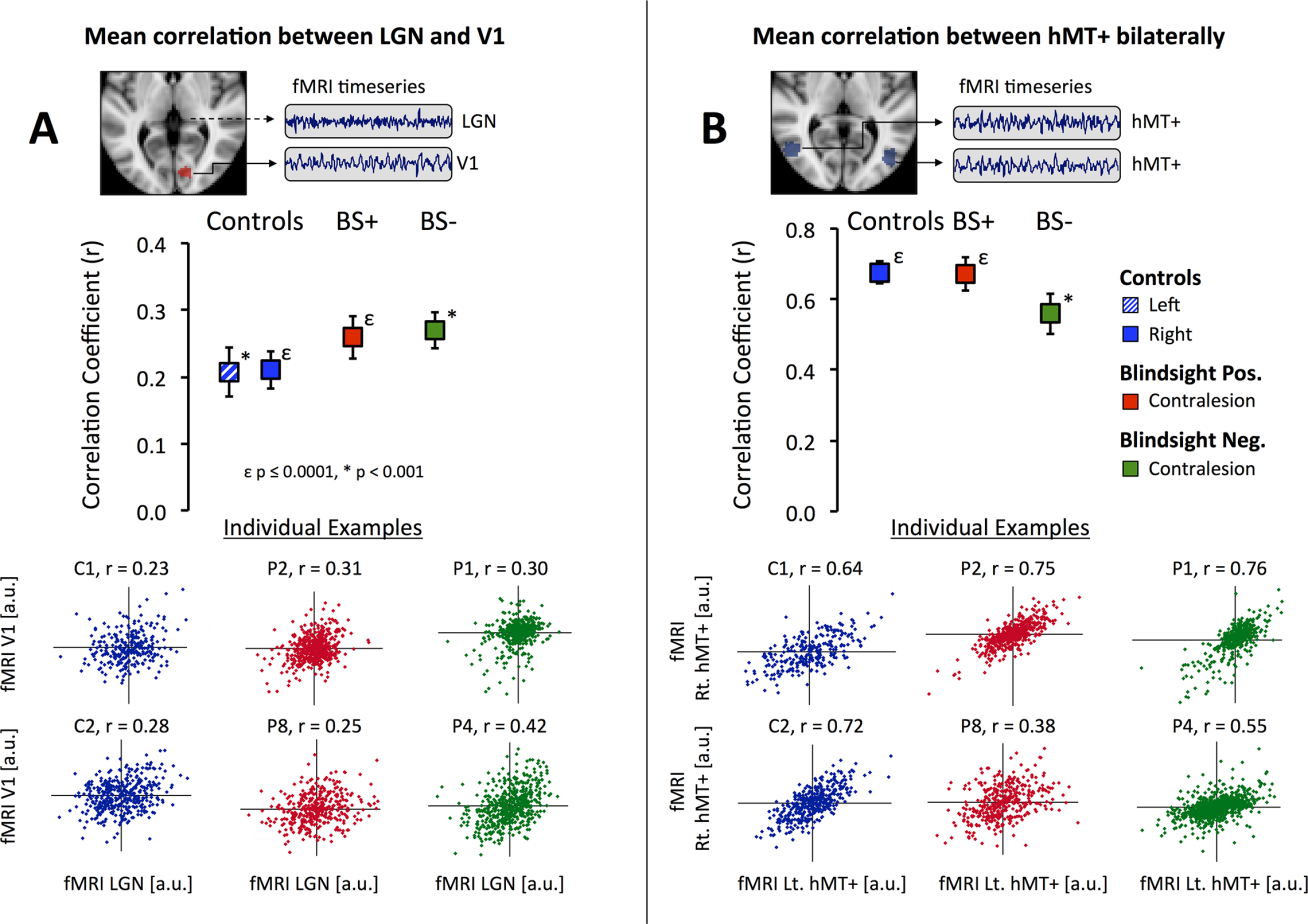
Functional connectivity of the visual pathways in patients and controls. **(A)** Correlation of LGN and V1 in the same (undamaged) hemisphere over the entire fMRI timeseries, after stimulus-evoked activity has been regressed out. **(B)** Correlation of hMT+ bilaterally. Box plots show Fischer-corrected mean correlation coefficients comparing participant group ± SEM. Statistical symbols represent significance levels for one sample t-tests against baseline (zero): ε *p* ≤ 0.0001, * *p* < 0.001. Scatterplots are individual examples of fMRI signal in ROI1 versus ROI2. Each point represents a single fMRI volume. Plots for patients in **panel A** are correlations in the contralesional hemisphere. BS+ is blindsight positive, and BS- blindsight negative. Underlying data can be found in S2 Data. fMRI, functional MRI; LGN, lateral geniculate nucleus; ROI, region of interest; SEM, standard error of the mean; V1, primary visual cortex.

LGN–V1 correlation in the nondamaged hemisphere (Fig 2A) was similar across all participant groups (F = 1.0, *p* = 0.4, df = 2). Bilateral hMT+ correlation was also very similar across all groups, indicative of preserved functional connection between hMT+ in patients irrespective of blindsight status (Fig 2B, F = 1.9, *p* = 0.2, df = 2).

**Fig 3:**
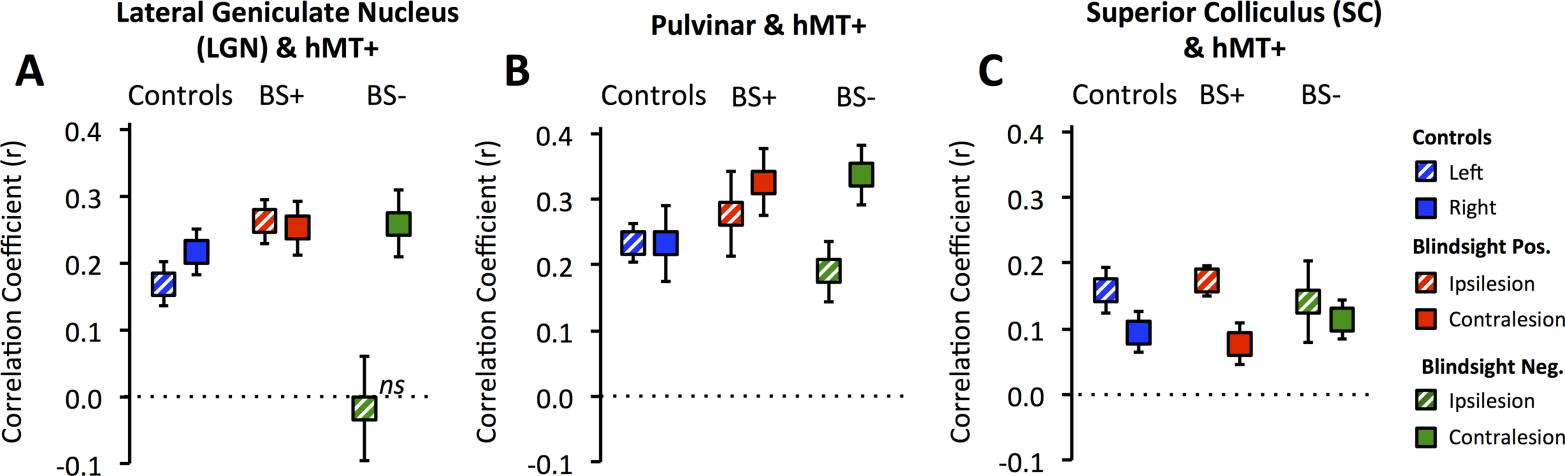
Functional connectivity of subcortical hMT+ pathways in patients and controls. **(A)** Correlation of LGN and hMT+. **(B)** Correlation of ventral pulvinar and hMT+. **(C)** Correlation of SC and hMT+. Box plots show Fischer-corrected mean correlation coefficients comparing each participant group ± SEM. Results are shown separately for the intact (solid boxes) and damaged hemispheres (striped boxes), and for left (striped) and right (solid) hemispheres in controls. Underlying data can be found in S3 Data. LGN, lateral geniculate nucleus; SC, superior colliculus; SEM, standard error of the mean.

There was a significant effect of participant group on LGN–hMT+ correlation (Fig 3A, F = 10.1, *p* = 0.001, df = 2). Pairwise analysis for the damaged hemisphere showed that blindsight-negative patients had a significantly lower mean correlation coefficient compared to blindsight-positive patients (−0.03 ± 0.08 SE versus 0.25 ±0.03 SE, *t* = 3.6, *p* = 0.003, df = 12) and remained at zero. In contrast, functional connectivity between hMT+ and ventral pulvinar (Fig 3B) or hMT+ and SC (Fig 3C) showed no effect of group (F = 0.5, *p* = 0.6, df = 2, for both ROIs) and no difference between blindsight-positive and -negative patients in the damaged hemisphere (pulvinar: *t* = 0.9, *p* = 0.4, SC: *t* = 0.5, *p* = 0.6, df = 12). Blindsight-negative patients also showed a hemispheric difference for LGN (*t* = 3.0, *p* = 0.01, df = 10) but not ventral pulvinar connectivity (*t* = 2.1, *p* = 0.06, df = 10).

**Fig 4:**
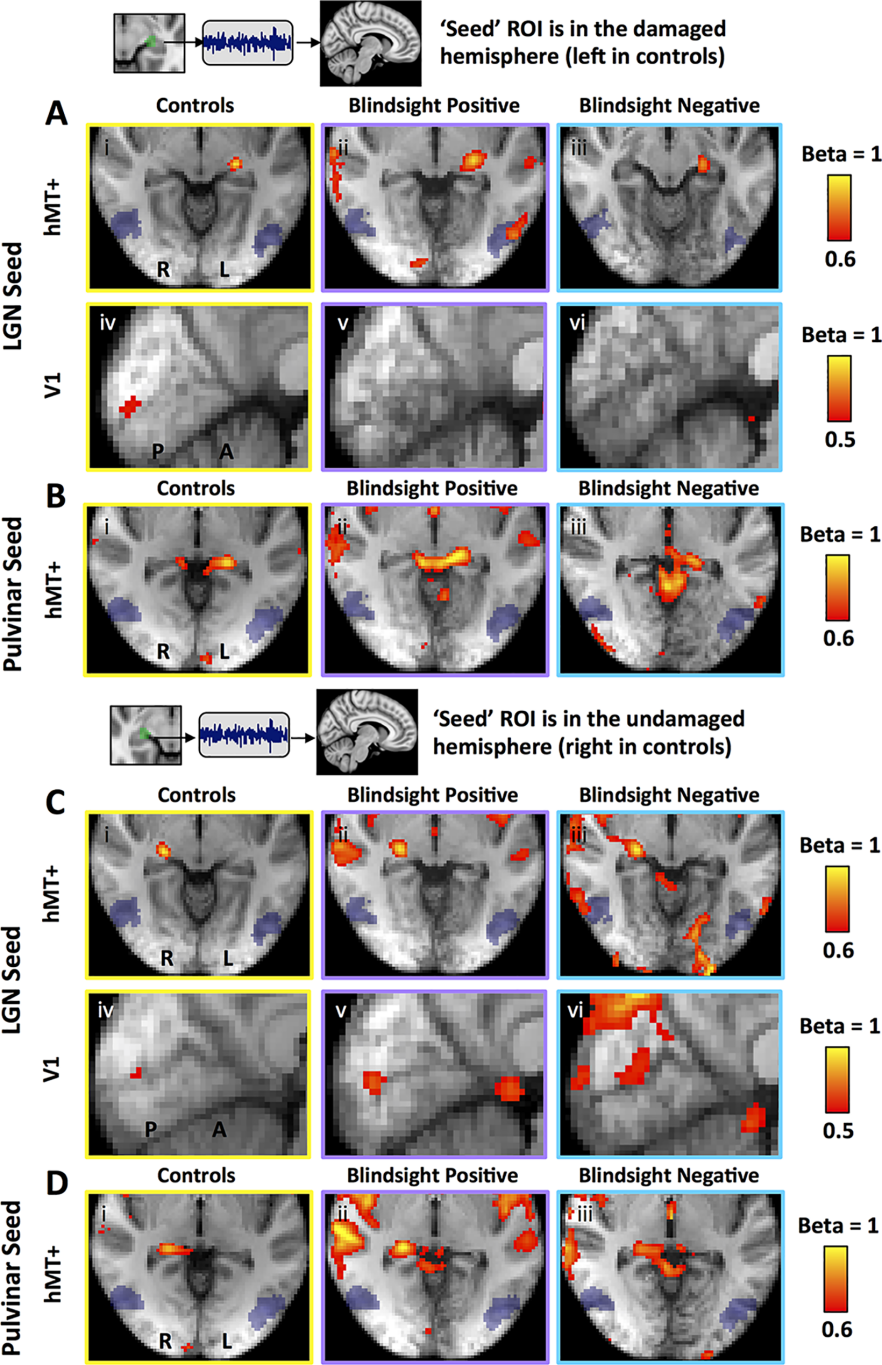
Seed region correlation maps for LGN and ventral pulvinar, in patients and controls. ‘Seed regions’ are **(A)** LGN in the damaged hemisphere (left in controls), **(B)** ventral pulvinar in the damaged hemisphere (left in controls), **(C)** LGN in the undamaged hemisphere (right in controls), **(D)** ventral pulvinar in the undamaged hemisphere (right in controls). Results are shown separately for controls (left column), blindsight-positive patients (middle column), and blindsight-negative patients (right column). Mixed effects analyses, displayed on average high-resolution structural scans transformed to MNI space (radiological convention). Shaded blue areas are binarized Jülich-defined probabilistic maps of hMT+. LGN, lateral geniculate nucleus; MNI, Montreal Neurological Institute.

This suggests that the key difference in functional connectivity between blindsight-positive and -negative patients was the presence of a functional connection between hMT+ and LGN in the damaged hemisphere. We performed an additional analysis without regressing out stimulus-evoked responses and found the same results (one-way ANOVA F = 4.3, *p* = 0.02, df = 2; paired *t* = 3.4, *p* = 0.005, df = 12), implying that in patients with blindsight this functional connection was also present during visual function. These analyses, however, have not evaluated whether differences in connectivity were specific to our predefined regions of interest or if they reflect a global process independent of the ROIs and our hypothesis. To address this, we performed an additional whole-brain mixed effects analysis measuring the voxels where neural activity most closely matched the time series of LGN, ventral pulvinar, and hMT+. This technique was used to generate seed region correlation maps, in which the ‘seed’ ROI should necessarily demonstrate a high correlation coefficient represented by a beta of 1 [12]. Co-active regions would similarly possess a high beta, with maps retaining a high spatial resolution since every voxel is tested [13]. As expected, group correlation maps showed a high beta in the ‘seed’ regions, reflecting consistency between subject-specific ROIs and their precise transformation to standard space (Fig 4). When LGN was the ‘seed’ region, control participants also demonstrated a relatively high beta in a small region of the calcarine cortex corresponding to retinotopically active V1 (Fig 4Aiv and 4Civ), reflecting the functional geniculostriate pathway. A similar region of calcarine cortex was co-activated in the undamaged hemisphere of patients when the seed was LGN in the same hemisphere (Fig 4Cv and 4Cvi). For LGN in the damaged hemisphere of both patient groups, there was no demonstrable V1 co-activation (Fig 4Av and 4Avi), likely reflecting the damage to that region (and/or its input). In contrast, blindsight-positive patients showed a relatively high beta in hMT+ of the damaged hemisphere compared to other participant groups and a small region of calcarine cortex in the undamaged side (Fig 4Aii).

In contrast to the LGN, when ventral pulvinar was used as the ‘seed’, all groups showed robust co-activity in the SC, an area known to share an important connection with the pulvinar [14,15]. There was also notable connectivity with V1 in the undamaged hemisphere (Fig 4D, [16]) but no major connectivity with hMT+ (Fig 4B).

**Fig 5:**
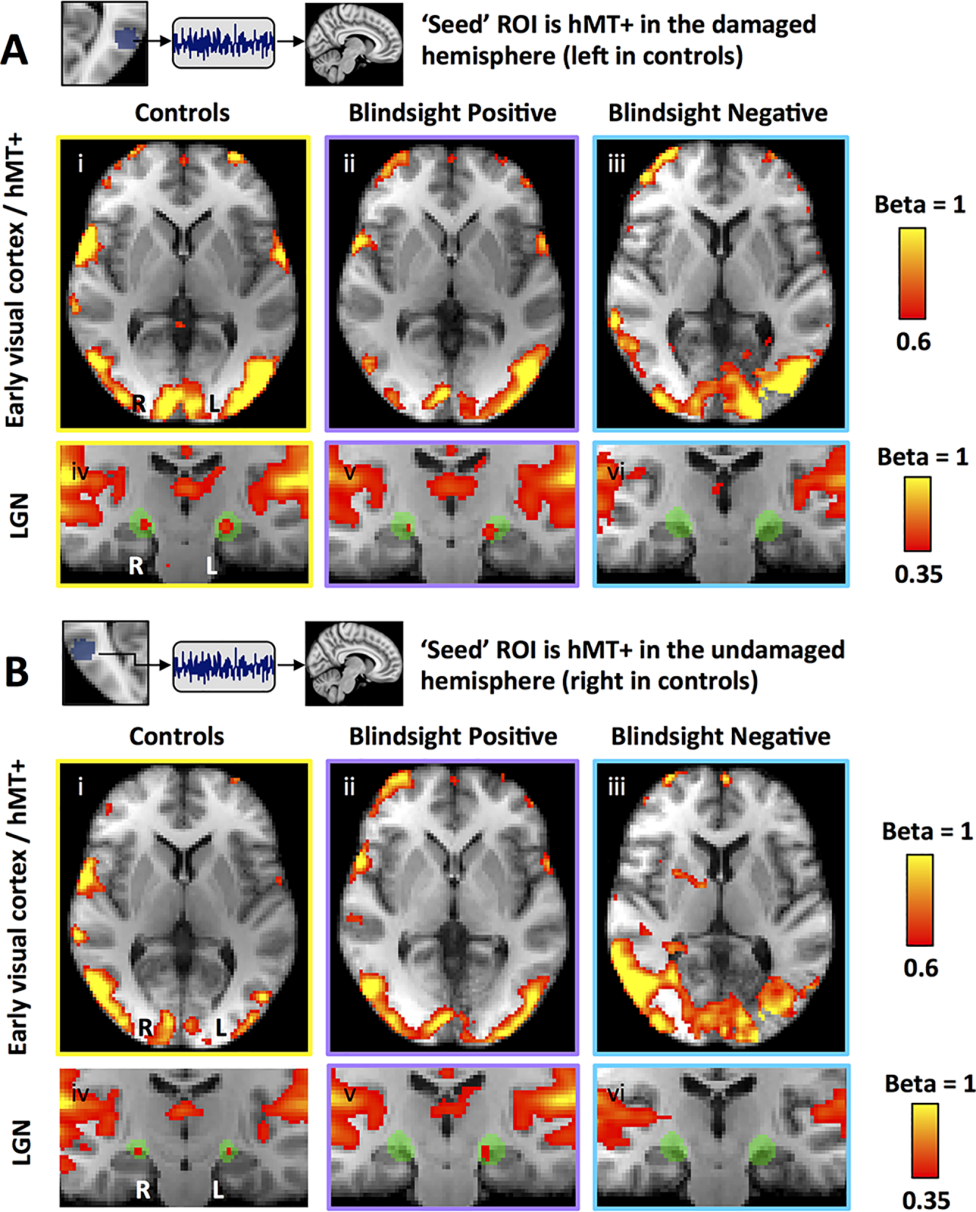
Seed region correlation maps for human motion area (hMT+), in patients and controls. ‘Seed region’ is **(A)** hMT+ in the damaged hemisphere (left in controls) and **(B)** hMT+ in the undamaged hemisphere (right in controls). Results shown separately for controls (left column), blindsight-positive patients (middle column), and blindsight-negative patients (right column). Upper rows show axial slices through early visual cortex and hMT+; lower rows show coronal slices through LGN. Mixed effects analyses, displayed on average high-resolution structural scans, transformed to MNI space (radiological convention). Shaded green areas are binarized Jülich-defined probabilistic maps of LGN. LGN, lateral geniculate nucleus; MNI, Montreal Neurological Institute.

When hMT+ was used as the ‘seed region’ (Fig 5), controls demonstrated marked functional connectivity throughout the visual cortex, including hMT+ in the opposite hemisphere and V1 in both hemispheres, consistent with previous reports [17]. In subcortical regions, connectivity was also demonstrable in the LGN bilaterally, albeit to a lesser extent (Fig 5A and 5B, left column). No equivalent connectivity was seen in the ventral pulvinar or SC, although these regions were co-activated in both hemispheres of all three participant groups if a slightly lower beta threshold of 0.32 was used (rather than 0.35). This pattern of connectivity was likely to reflect the major visual pathway and its rich network of intra- and interhemispheric connections. A very similar pattern was demonstrated in blindsight-positive patients, except for a relatively low beta in calcarine cortex of the affected hemisphere, reflecting the region of tissue damage. This was less apparent in blindsight-negative patients. Blindsight-negative patients also showed relatively poor connectivity with LGN, particularly in the damaged hemisphere, where ipsilateral geniculate co-activity was not demonstrable even when a low threshold was applied (beta > 0.2).

### Behavioural–neuroimaging comparisons

Of the eight blindsight-positive patients, only half could discriminate direction of motion above chance. We were interested in whether this subgroup showed greater BOLD activity and/or connectivity compared to blindsight-positive patients who were unable to discriminate motion direction. Indeed, these four patients did show slightly stronger motion responses in hMT+ (0.3 ± SE 0.13 versus 0.1 ± 0.07, *t* = 1.6, *p* = 0.2, df = 6), as well as slightly greater LGN/hMT+ functional connectivity (0.27 ± 0.05 versus 0.22 ± SE 0.06, *t* = 0.6, *p* = 0.5, df = 6), although the differences were relatively small and nonsignificant.

To investigate the behavioural–neuroimaging association further, we performed a correlation analysis between mean behavioural performance in both experiments and fMRI activity and connectivity across all patients (*n =* 14). hMT+ motion responses showed a weak but positive correlation with behavioural performance (r = 0.38, *p* = 0.18), as did LGN–hMT+ connectivity (r = 0.38, *p* = 0.18). There was also a positive but weaker correlation between behavioural performance and pulvinar–hMT+ (r = 0.29, *p* = 0.31) or SC–hMT+ connectivity (r = 0.21, *p* = 0.47). More notable was a significant correlation between behavioural performance and the ratio of blind to sighted fMRI responses in hMT+ (r = 0.62, *p* = 0.018). In other words, patients with the strongest hMT+ activity for motion in the blind field relative to their sighted field performed best at behavioural assessments of blindsight using the same visual stimuli.

### Relating lesion size to behavioural performance and functional connectivity

In order to determine the extent to which blindsight performance and the underlying neural mechanisms relate to the size of the lesions, we quantified the damage in each patient. Lesion size did not differ significantly between blindsight-positive and -negative groups (*t* = 1.5; df = 12; *p* = 0.16), although blindsight patients on average had smaller lesions (13,693 mm^3^ ± 1,825 mm^3^ SEM) than those without blindsight (21,212 mm^3^ ± 5355 mm^3^ SEM). This is illustrated by the summed lesion masks for each patient group on a standard space template (S2D and S2E Fig) and the individual lesion maps in structural space (S4 Fig). Reflecting the small difference in lesion size between the two patient groups, there was a moderate inverse relationship between lesion size and behavioural performance across both tasks (r = −0.48; *p* = 0.08). However, there was no relationship between lesion size and functional LGN–hMT+ connectivity (r = 0.21, *p* = 0.5), or hMT+ signal change (r = −0.24, *p* = 0.4). Thus, lesion size did not appear to be a critical factor in determining neural response.

Aside from lesion size, the degree to which lesions involve hMT+ and its innervating connections would also be critically important. Lesion maps (S4 Fig) suggest that the lesion of one blindsight-negative patient (P12) did encroach upon hMT+, while it appeared intact in the other five blindsight-negative patients, including its afferent white matter. P1 also had a large lesion extending to the posteromedial border of hMT+; however, this should not have impacted upon the subcortical or interhemispheric white matter connections (see S4 Fig for map). Accordingly, we quantified lesion involvement of hMT+ and its surrounding white matter in each patient (S5 Fig). We found no difference when comparing blindsight-positive and -negative patients (*t* = 1.7, *p* = 0.11, df = 12), and hMT+ lesion size showed no association with LGN–hMT+ functional connectivity (*t* = 1.6, *p* = 0.13, df = 13), hMT+ activity (*t* = 1.4, *p* = 0.19, df = 13), or behavioural performance (*t* = 1.6, *p* = 0.13, df = 13) in paired *t* tests using lesion size as the dependent variable.

When recruiting a relatively large group of patients, it is challenging to ensure a completely homogenous lesion pattern. However, any specific differences in lesion size or extent can be informative. P12, whose lesion may have encroached on hMT+, demonstrated weak functional connectivity (outside one SD of the mean) in all three subcortical pathways (pulvinar–hMT+: r = 0.004, SC–hMT+: r = −0.13) and between hMT+ bilaterally (r = 0.33), and this contributed to slightly lower averages for pulvinar, collicular, and interhemispheric (but not LGN) connectivity in blindsight-negative patients (see Fig 2B and Fig 3B and 3C). The variability amongst naturally occurring human V1 lesions has been highlighted as a limitation of human research compared to nonhuman primates [18]. It certainly emphasizes the limitations in carrying out individual case studies, which have predominated in the blindsight literature over the last several decades. However, the heterogeneity in the precise location of structural damage can also be extremely useful and has permitted patients to be classified according to their distinct residual visual performance. By determining which connections and characteristic fMRI responses are consistent amongst blindsight-positive and -negative patients, it may be possible to identify which underlying structures and pathways are involved.

To summarise our results, blindsight-positive patients showed (i) significant neural activity in hMT+ to motion stimuli in the ‘blind’ visual field, (ii) a relatively preserved response to speed in hMT+, and (iii) a correlation between resting BOLD signal in LGN and hMT+ in the damaged hemisphere. None of these findings were demonstrable in patients without blindsight. However, blindsight-negative patients did show functional connectivity between hMT+ and (i) SC and (ii) ventral pulvinar that was no different to healthy controls or patients with blindsight, suggesting that the LGN has a specific functional role in blindsight.

## Discussion

This study tested a group of patients, all of whom sustained unilateral damage to V1 in adulthood. Critically, not all the patients demonstrated the same residual visual function, allowing us to relate brain activity to blindsight performance using the same visual stimuli. At the most basic level, we have shown that human motion area hMT+ does not require V1 to demonstrate a normal fMRI response to speed. More importantly, we have found additional evidence to support a specific functional role for the LGN in blindsight and to suggest that functional connectivity with the ventral pulvinar and SC is not sufficient for blindsight.

Intrinsic functional connectivity corresponds to a number of anatomical markers of connectivity, including anatomical traced pathways [19], axonal pathway density [20], and intracortical myelin content [21]. There is also evidence that the strength of correlation between functionally coupled regions relates to measures of cognition and behaviour and can be useful markers of brain system integrity in neurological and psychiatric disorders (e.g., see [22] for review). When two regions, such as left and right motor cortices, share a high correlation coefficient (r = 0.8, [23]) similar to left and right hMT+ in our study, this is interpreted as an example of strong functional interconnectivity. In contrast, a correlation coefficient of zero between LGN and hMT+ in blindsight-negative patients is similar to the correlation between motor and visual cortices in healthy individuals and implies a weak or absent functional connection [23]. The relationship between functional and structural connectivity measures is important to consider, as these can give different results. It is possible for normal-appearing functional connectivity to occur even when there is a known disruption in the physical connection [24], perhaps explained by intact polysynaptic anatomic connections or common feed-forward projections [25]. In previous work using diffusion MRI, we could only identify streamlines between SC and hMT+ in 7 out of 9 healthy controls and 14 out of 17 patients (in the intact hemisphere) [3]. In the current study, we observed intact functional connectivity for this pathway in all participants. This is an important discrepancy, as we cannot be certain which methodology reflects the underlying physiology most accurately. It may be possible for a functional connection to exist between SC and hMT+ via an indirect pathway while direct, diffusion MRI–traceable connections were absent. In support of this, the connection between SC and hMT+ is believed to relay via a synapse in the pulvinar nucleus [26]. Another explanation for the lack of consistency in diffusion tractography was that the size of the tract rendered it difficult to trace, and therefore, differences in brain geometry had a greater effect on identification. These points emphasise the importance of functional connectivity as a physiological measure of connected brain regions and the limitation of using diffusion MRI tractography alone.

It is well described that hMT+ remains active after V1 damage in blindsight [27–31]. More recently, we have started to explore how hMT+ responds to common stimulus parameters after V1 is damaged [32,33]. We have previously reported that normal hMT+ responses to global motion [32] and contrast sensitivity [33] are dependent on V1, as both become abnormal after unilateral V1 damage, even in the presence of blindsight [33]. The current study, however, has shown that a classical hMT+ response to speed remains intact in patients who demonstrate blindsight. This is consistent with the recent finding in healthy individuals that visual motion information from LGN can reach hMT+, bypassing V1, in response to both slow and fast speeds of motion [34]. There are also two reports in the literature that have measured macaque middle temporal area (MT) neuronal responses to speed after striate cortex removal, and both imply that speed tuning may be retained, including responses to slow motion [35,36]. Together, these results suggest that visual input from regions other than V1 are sufficient for normal patterns of response to speed, but only in patients demonstrating blindsight.

The LGN is often implicated in blindsight as a possible nonstriate source of input to the extrastriate cortex [3,37,38]. Neuroanatomical studies suggest that there is a direct connection between the LGN and extrastriate cortex [39,40], including specific projections to middle temporal area (MT) [41–43]. However, its relatively small size and deep location makes it particularly challenging to image [44]. Our results support a functional role for the LGN in blindsight for several reasons. Firstly, blindsight-positive patients showed retained functional connectivity between LGN and hMT+, whilst this was absent in blindsight-negative patients. This was demonstrated both at a whole-brain level and using an ROI analysis. Secondly, these results were specific to the LGN, as blindsight-negative patients showed retained functional connectivity with both ventral pulvinar and SC that was no different to healthy controls or patients with blindsight, despite both structures being implicated in blindsight [5,8]. We are confident that we were able to distinguish the LGN from the ventral pulvinar since the subcortical regions co-activating with hMT+ showed a strong correspondence to Jülich probabilistic maps of the LGN using whole brain analyses (Fig 5Av) [45]. Similarly, seeding in the pulvinar (but not the LGN) showed robust co-activation with SC throughout all participant groups.

One limitation was that directionality of the functional connections could not be determined. Theoretically, hMT+ in blindsight could be driven by non-geniculate input but show intact functional connectivity with LGN due to retained feedback [46]. On balance, this seems unlikely, as in blindsight-negative patients, the LGN was the only subcortical region to lose a functional connection with hMT+. Connectivity with the undamaged hemisphere was also no different to controls, consistent with our recent report of a patient with bilateral V1 damage [47]. In any case, it is suggested that feedback projections from MT to LGN may require V1 [46]. A further challenge in this study was the relatively noisy subcortical signal, which was susceptible to motion artefact. This was, however, relevant to all subcortical regions, and our measures of functional connectivity proved robust and consistent between individuals.

The role of the pulvinar in residual vision and blindsight has been emphasized after identifying a direct connection with MT in monkeys [24,48,49], particularly since the connection between the medial division of the inferior pulvinar and MT is enhanced following early lesions of V1 [8]. The current study used a ventral pulvinar mask consistent with the literature [50]. We also employed whole-brain analyses to determine which structures showed functional correlation with hMT+. The role of the pulvinar therefore requires further investigation, as it may not carry sufficient information to facilitate significant behavioural performance in the tasks used here. However, it was interesting that blindsight-negative patients showed weak hMT+ activity to slow motion (4°/s, Fig 1Biv). Perhaps such an abnormal speed response could be supported by a functional connection with the pulvinar and/or SC, which appeared to be intact in the blindsight negative group. Macaque MT neurons show a variety of speed-tuning curves that include the pattern seen in blindsight-negative patients (with peak responses at 4°/s) and more classical response with peaks at 8 or 20°/s (e.g., [51,52]). The underlying basis for these distinct neuronal subsets is unknown. One possibility is that they reflect different inputs, e.g., from intact V1, or direct subcortical nuclei such as LGN, SC, and pulvinar. V1 normally exhibits a similar preference for slow speed, with a negative slope similar to hMT+ in blindsight-negative patients [53]. Inferior pulvinar neurons also possess a range of speed-sensitivities, mostly preferring slower motion under 32°/s [54,55], not dissimilar to V1 responses. It is suggested that those responses may arise from striate cortex inputs [56], and it was interesting that blindsight-negative patients showed evidence of peripheral V1 activation, making this a possible source of input. Macaque LGN neurons also demonstrate a range of speed preferences, particularly in magnocellular neurons, but most prefer somewhat faster motion [57]. The observation that blindsight-positive and -negative patients showed a different speed-tuning curve in hMT+ despite equivalent preservation of hMT+ and its surrounding white matter suggests that speed-tuning is unlikely to arise de novo in hMT+ but is perhaps determined by the innervating pathway and its subcortical structures.

It is also possible that early and late V1 lesions result in different pathways carrying residual visual function. All the patients in this study acquired V1 lesions in adulthood. It would be useful to compare to patients with congenital or early life lesions, such as the recent case of a child with extensive damage to the occipital lobe bilaterally who showed preserved visual function [9]. This child showed a reduction in streamlines between LGN and hMT+ measured with diffusion imaging but showed an increase between the medial portion of the inferior colliculus and hMT+ in one hemisphere.

A small number of studies have implicated the SC in ‘non-aware’ or indirect blindsight [5,58,59]. However, those patients sustained brain injury at birth or in early childhood. After striate cortex removal in the macaque, directionally selective responses in MT stop if the SC is additionally destroyed [35,60], while there is no effect resulting from the removal of the SC alone. Behaviourally, monkeys who retain saccadic eye movements towards a target in their blind field after a V1 lesion lose this ability and the potential for recovery if the ipsilesional SC is also inactivated [61,62]. These findings could be reconciled with studies implicating the LGN in blindsight if the SC provides input to LGN in a collicular–geniculate–extrastriate pathway [37]. Indeed, Kato and colleagues [62] suggest two complementary pathways, one collicular–geniculate-hMT+ and the other collicular–pulvinar–hMT+, which could be consistent with our measures of functional connectivity between these structures and the distinct hMT+ response pattern in blindsight-positive and -negative patients.

Lastly, behavioural sensitivity to speed showed an ‘inverted-U’ pattern that was consistent with previous reports [63]. This was similar to characteristic speed responses measured in hMT+ using fMRI [53,64]. Patients also demonstrated a linear relationship between blindsight performance and relative hMT+ activity, which extends previous reports simply categorising patients with or without functional activity and significant accuracy [65]. This has important implications for rehabilitation, as it may be that enhancing this residual activity and functional connection could improve detection and discrimination performance.

### Conclusions

In summary, we identified a functional connection between LGN and hMT+ in patients with blindsight that was absent in patients without blindsight, despite a retained functional connection with ventral pulvinar and SC. This supports a critical functional role for the LGN in human blindsight, and in particular its connection with hMT+, reinforced by recent evidence for an intact anatomical connection between these structures [3]. Our results also revealed that hMT+ does not require intact V1 for a normal speed response, although it does require a functional connection with the LGN. This suggests that the LGN may support motion-selective input to hMT+ in the absence of V1. These results focus on behavioural and neural responses to visual motion, which is a critical component of blindsight (see [66] for recent review). In future work, it will be necessary to explore how such pathways interact with other aspects of blindsight function and whether distinct tasks or stimuli might engage separate mechanisms in the absence of V1.

## Materials and methods

### Participant details

Fourteen patients with adult-onset unilateral V1 damage took part in this study (see S1 Table for details). The location of any additional non-V1 damage is shown in S2D and S2E Fig, and S4 Fig. No patients sustained damage to subcortical structures, including the LGN and pulvinar. Average age at the time of participation was 55.6 years ± 15.2 SD; average time after pathology onset was 49 months (6–252 months). Eight age-matched, healthy participants (50.1 ± 14.6 SD years) served as controls. Written informed consent was obtained from all participants, and ethical approval was provided by the Oxford Research Ethics Committee (Ref B08/H0605/156). All experiments adhered to the Declaration of Helsinki.

### Stimuli and experimental design

Visual stimuli were generated using MATLAB (Mathworks) and the Psychophysics Toolbox [67,68]. Each dot was 0.075° in diameter and had an infinite lifetime, with an average dot density of 8 dots/°^2^. Visual stimuli consisted of an aperture of 5° or 8° diameter containing static or coherently moving black dots (luminance 0.5 cd/m^-2^) at 4, 8, 20, or 32°/s on a uniform grey background of luminance 50 cd/m^-2^. Stimuli were positioned inside a region of dense visual field loss in patients a minimum of 2.5° from fixation (S6 Fig). The extent to which stimuli covered the scotoma (as a percentage) was estimated for each patient from the Perimetry Visual Field Index (VFI), provided in S6 Fig. Stimulus size and position was matched as closely as possible in eight age-matched controls (S2A Fig), with no significant difference in distance between fixation and stimulus edge (x or y coordinates) when comparing patients to controls (mean x = 3.6 ± 0.30 SE patients versus 3.8 ± 0.4 SE controls, *t* = 0.4, *p* = 0.7, df = 20; mean y = 0.65 ± 0.66 SE patients versus 0.49 ± 0.68 SE controls, *t* = 0.2, *p* = 0.9, df = 20). Stimuli in blindsight-negative patients were also no deeper into the visual field than blindsight-positive patients (stimulus edge 4.0° ± SD 0.9 in blindsight-positive versus 3.5° ± SD 1.2 in blindsight-negative patients, *t* = 0.9, *p* = 0.4, df = 12).

To select the stimulus location in patients, we required a perimetry threshold *p* < 0.005 or < −20dB (which ever was more stringent) for pattern deviation compared to age-matched controls at the stimulus location. This meant that the patients in our study were unable to see even the brightest unattenuated stimuli at that location in the visual field. To verify that we had not inadvertently chosen locations in blindsight-positive patients that were more sensitive than those in blindsight-negative patients, we calculated the average pattern deviation by taking the value closest to the stimulated location using Humphrey Perimetry. The residual visual sensitivity was no different in the two groups (−32.8dB ± SE 0.8 blindsight positive versus −33.2dB ± SE 0.9 blindsight negative, *t* = 0.3, *p* = 0.8, df = 12).

Outside the scanner, two behavioural experiments were performed: (1) 2AFC temporal detection and (2) 2AFC direction discrimination (S1 Fig). The experiments were conducted on the same day as scanning, using a 60-Hz CRT monitor at a viewing distance of 68 cm. Throughout behavioural experiments, participants were asked to maintain fixation, with the investigator observing this in real time using an Eyelink 1000 Eye Tracker (SR Research Limited, Ontario, Canada). Anyone making even a small eye movement into their damaged hemifield was given specific instruction not to do so, and it was explained that these data would have to be discarded.

At the start of the experiment, an identical, static test stimulus was used to confirm that patients were unable to see the stimulus at its selected size and location in the visual field. This was done using a predicted aperture size and locus based upon prior perimetry results. Stimulus location had to be restricted to the boundary of the fMRI display, which subtended 23° horizontally and 13° vertically. This influenced whether a 5° or 8° diameter stimulus was chosen, as the stimulus had to stay inside the ‘blind’ field while remaining on screen. The stimulus of choice was an 8° diameter aperture, but if this was not possible, the stimulus was reduced to 5° diameter. If the criteria were unachievable using either stimulus size, the patient was excluded from the study. If the patient was able to see any part of the test stimulus whilst fixating on the central cross, the aperture was repositioned 0.5° deeper into the scotoma (according to the Perimetry report) until the patient could no longer see any part of the stimulus at all. Any trials with eye position more than 1° from fixation were excluded from analysis.

#### Experiment 1: 2AFC temporal detection

Patients were asked to indicate whether a stimulus appeared in the first or second time interval using a two-alternate forced choice paradigm (S1A Fig). If they saw nothing, they were instructed to guess. Onset of each interval was indicated by a 500-ms auditory tone, with 300 Hz marking onset of the first interval and 1,200 Hz marking the onset of the second. Visual stimuli appeared for 500 ms with jittered onset while the participant fixated on a central black cross. The allocated interval (first or second) was generated at random. Stimulus speed was altered parametrically between the five conditions at random, with an average of 20 trials per condition.

#### Experiment 2: 2AFC direction discrimination

Patients were asked to indicate whether motion direction was horizontal or vertical (S1B Fig). Again, if they saw nothing, they were instructed to guess. Visual stimuli appeared for 500 ms with jittered onset whilst the participant fixated on a central black cross. Stimulus speed was altered parametrically between the four motion conditions at random, with an average of 20 trials per condition.

### Blindsight definition

The presence or absence of residual visual function (blindsight) was determined according to patients’ ability to detect stimuli above chance, i.e., Experiment 1. Specifically, this was defined as achieving either an average score or a score for individual conditions that was significantly above chance, using a statistical threshold of *p* < 0.05 and a cumulative binomial distribution. This was an identical method to our previous work, except that the stimulus was moving dots rather than a drifting Gabor [33].

We selected stimulus location based upon perimetry results, as detailed above. Necessarily, this meant that all patients showed the same abnormal visual performance for their test locations. Aside from demonstrating the same visual sensitivity on perimetry, stimuli in blindsight-negative patients were no deeper into the visual field than they were for blindsight-positive patients, suggesting this was not a critical factor for the difference in behavioural performance.

Previous blindsight studies have employed a variety of visual stimuli (moving dots, gratings, moving bars, high luminance targets [31,37,63,69]) and a number of different techniques for assessment, including 2AFC, indirect behavioural performance, saccadic eye movements, and navigational performance (e.g., [5,70–72]). It is also common to target only one retinal location in blindsight testing [31,37,63,69,70]. The critical point for the definition of blindsight is that patients show significant performance despite absent visual capacity in the targeted region of the visual field. We ensured that a conservative threshold was used to target truly ‘blind’ regions of scotoma, which we demonstrated to be no different in patients with or without blindsight. Since we were particularly interested in the role of hMT+, we assessed whether a moving stimulus could be detected without awareness for our definition of blindsight.

Using these criteria, eight patients were categorized as ‘blindsight positive’, as they could detect the stimulus inside their blind hemifield significantly above chance (P2, P3, P5, P8, P10, P11, P13, P14). Of these individuals, four could also discriminate motion direction above chance (Experiment 2; P5, P8, P10, P14). With regard to subjective awareness, only two patients reported any awareness of the stimuli during the experiment (P3 and P10). Both had been completely unaware of static dots in the pre-experiment assessment, in which static dots were positioned at the same coordinates, without fast onset/offset. For moving stimuli, P10 reported knowing that something was there, but was unable to distinguish what it was. P3 also could not describe what she saw, suggesting she had been looking at ‘streaks or shadows’. Both patients were at ceiling on the detection task, but P3 remained at chance on the direction discrimination task.

Of note, five of the patients (P3, P8, P10, P11, P13) took part in a previous fMRI study [33], in which they also demonstrated significant blindsight performance for detection of a drifting Gabor.

### Behavioural eye-tracker results

Eye movements were defined as a change in fixation towards the scotoma of 1 degree or more. This would capture all eye movements irrespective of their type, i.e., saccadic, slow drift, nystagmus. The threshold of 1 degree ensured that stimuli could never be directly fixated but would always remain inside the scotoma. Although microsaccades were possible, these would not bring the visual stimulus into the seeing portion of the visual field. This methodology was the same as previous work (Fig 2B in [33]), in which we also provided examples of successfully identified saccades. Seven trials were removed from analysis in Experiment 1 and 2 trials from Experiment 2 due to eye movements of more than 1 degree towards the stimulus calculated from retrospective eye tracker data analysis. At the time of the experiment, a further 6 trials were flagged for exclusion in Experiment 1 and 4 in Experiment 2 due to real-time observation of the experimenter or feedback from the patient. In total, this accounted for 0.93% of trials in Experiment 1 and 0.54% of trials in Experiment 2 that were excluded from analysis due to inappropriate eye position.

### fMRI procedure

The same stimuli were viewed during fMRI, presented separately to each hemifield. Stimuli during scanning were presented on a 1,280 × 1,040 resolution monitor at the back of the MRI scanner bore. Participants viewed stimuli via a double mirror mounted on the head coil. When in position, the screen subtended a visual angle of 23° × 13°. The same 5 speed levels were presented separately to each hemifield, representing a 10-condition block design (S2B Fig). For each block, the aperture of moving or stationary black dots appeared for 16 s. Direction coherence was 100%, and dots moved at a constant speed. Angle of drift changed at random every two seconds from a choice of 8 directions. A 10-s rest period followed each block. Throughout all experiments, participants performed a task to maintain fixation by pressing a button every time a central fixation cross changed colour from black to red (S2B Fig). Colour changes occurred at random, lasting 300 ms in duration, and participants were instructed at the start to try not to miss any red crosses. It was emphasised that they must try to maintain fixation throughout and avoid moving their eyes around the screen. An EyeLink 1000 eye tracker (SR Research Limited, Ontario, Canada) was again used to confirm central fixation by recording eye position (see section fMRI eye tracking).

### MRI acquisition and preprocessing

Scanning took place using a 3T Siemens Verio MRI scanner at the Functional Magnetic Resonance Imaging Centre of the Brain (FMRIB, University of Oxford). At the start of each sequence, magnetisation was allowed to reach a steady state by discarding the first five volumes, an automated feature of the scanner. T2*-weighted EPI volumes covered 34 sequential 3-mm slices (repetition time, TR 2000 ms; echo time, TE 30 ms) with three runs, each lasting 260 s. In a single session lasting 13.2 min, 396 functional volumes were acquired. For one participant (P4) we collected one additional session of fMRI data. For three patients, we collected four runs of data (i.e., 526 volumes, P1, P2, P3). For one patient (P7) and one control, we only collected two runs of data, i.e., 266 volumes (8.9 min).

We also acquired a high-resolution (1 mm^3^) whole-head T1-weighted MPRAGE anatomical image (TE 4.68 ms; TR 2040 ms; flip angle, 8°) and a field map (TE1, 5.19 ms; TE2, 7.65 ms; 2 mm3) for each participant.

### Quantification and statistical analysis

fMRI preprocessing and statistical analyses were carried out using tools from FMRIB's Software Library (FSL, www.fmrib.ox.ac.uk/fsl). Non-brain tissue was excluded from analysis using the Brain Extraction Tool (BET) [73], motion correction was carried out using MCFLIRT [74], and images were corrected for distortion using field maps. For cortical ROIs and group contrast maps, spatial smoothing used a Gaussian kernel of FWHM 5 mm, and high-pass temporal filtering (Gaussian-weighted least-squares straight line fitting, with sigma = 13.0 s) was applied. For all subcortical ROI analyses, no spatial smoothing was applied to ensure that signals were not contaminated with adjacent structures. Functional images were registered to high-resolution structural scans using FLIRT [75] and to a standard Montreal Neurological Institute (MNI) brain template using FLIRT and FNIRT [76]. This enabled us to transform anatomical and probabilistic regions of interest into functional space for analysis (see S3 Fig for an illustration of subcortical ROIs in functional, structural, and standard space for each participant).

### fMRI eye tracking

Eye movements during fMRI can be a legitimate concern when considering results for visual stimulation inside a scotoma. In this study, three main lines of evidence suggest that this was not a problem and could not have accounted for the results. First, concurrent eye movement data was collected on most patients using an eye tracker positioned at the base of the MRI bore (*n* = 10). All of these patients underwent successful eye-tracker calibration, with accurate data throughout fMRI runs. For this group of patients, the mean number of eye movements was 5.8 ± 3.5 SEM, defined as a movement of 1.5 degrees or more towards the scotoma. This accounted for <0.3% of the scan duration, suggesting that any effects on the results are likely to be negligible. To confirm this, when scanner volumes corresponding to eye movements were regressed out of analyses, the results remained unchanged (r > 0.99). For the patients without eye movement data, there had been difficulty either with calibration due to their dense field loss or with visualisation due to the presence of corrective acuity lenses. In those situations, direct visualisation was used via video recording of the pupil to observe any overt eye movements during the experiment.

Second, participants performed over 90% on a concurrent behavioural task that required fixation throughout the experiment (S2C Fig). Brief colour changes of the fixation cross (300-ms duration) occurred at frequent and random intervals, and participants were given a window of 1 s to press a button connected to the stimulus computer via a parallel port, being specifically instructed not to miss any red crosses or move their eyes around the screen. In addition, before the fMRI scan, all participants took part in behavioural testing lasting at least 60 min, focussed on their damaged region of vision. Participants became very experienced at maintaining fixation during this assessment.

### Regions of interest

hMT+ masks were derived from probabilistic maps (Jülich atlas implemented in FSL) [77,78]. These were transformed into functional space for patients and controls to ensure consistency between participant groups. V1 masks in controls and in the undamaged hemisphere of patients were functionally defined so that they corresponded to stimulated regions of calcarine cortex. In native space, average hMT+ ROI volume was 94.8 ± 35.2 SD voxels in patients and 100.9 ± 42.0 SD voxels in controls (*t* = 0.5, *p* = 0.6, df = 42). Average V1 ROI volume was 16.2 ± 7.5 SD voxels in patients (undamaged hemisphere) and 24.4 ± 7.1 SD voxels in controls (averaged across hemispheres), the small volume reflective of the small 5°- or 8°-diameter stimulus used.

For the LGN and SC, binary masks were created by manual inspection and drawing over the anatomical T1-weighted images [79], using a radiological brain atlas to aid identification of landmarks (See S3 Fig for masks in all patients). The average LGN volume in patients measured 248 mm^3^ in the right and 246 mm^3^ in the left. In controls, average LGN volume was 240 mm^3^ in the right and 239 mm^3^ in the left. The average SC volume in patients was 195mm^3^ in the left and 177 mm^3^ in the right. For the ventral pulvinar, binary masks were created in MNI152 standard space according to the description of Arcaro and colleagues [50]. It was possible to visualise the nucleus as a region of low T1 intensity relative to surrounding tissue in the posterior most part of the thalamus. Masks were transformed to anatomical and functional space for each participant and were manually inspected to ensure accuracy (S3 Fig). Average ventral pulvinar volume was 385 mm^3^ in the left and 365 mm^3^ in the right. There were no significant differences in ROI volume between blindsight-positive and -negative patients.

### Lesion masks

Lesion masks were drawn manually in structural space (S4 Fig). For S2D and S2E Fig, these were nonlinearly transformed to standard space and binarised before being summed. To assess whether lesions encroached upon hMT+ and/or its surrounding white matter, we created subject-specific cuboidal ROIs that were centred on the ‘centre of gravity’ of the ipsilesional hMT+ ROI in structural space. The isotropic cubes measured 40 × 40 × 40 mm^3^, thus containing 64,000 1-mm^3^ voxels (see S5A and S5B Fig for examples in P6 and P12). We superimposed the binarized lesion mask over the cuboidal mask and counted the number of voxels that overlapped (red voxels in S5B Fig). The voxel counts are shown in the table in S5 Fig.

### fMRI analysis

All graphs, signal change calculations, and correlation statistics were calculated using data from participants’ native space.

For region of interest analysis, each experimental condition (e.g., left hemifield, 8°/s speed) was entered into the general linear model as a separate explanatory variable and was contrasted against the baseline fixation task to generate contrast of parameter estimates (COPEs) for each condition in every voxel. Signal change was then extracted from regions of interest within functional-space for each individual. The percentage of signal change was calculated by scaling the COPE by the peak-peak height of the regressor and dividing by the mean over time. These measures were averaged across participants to generate group plots for signal change as a function of the condition under investigation and were used in all correlation and regression analyses.

For whole-brain group analyses (Figs 4 and 5), it was necessary to align patient brains to a uniform pathological template with lesions located in the same ‘left’ hemisphere corresponding to a ‘right-sided’ visual field deficit. This required that the structural and functional images of three patients (P3, P5, P12) be flipped in the horizontal plane. All activation coordinates and images were in MNI space, with beta values displayed on mean structural images for the group transformed to standard space.

For the whole time series analyses, a value for residual BOLD signal in the ROI was obtained for each volume, and this was plotted against time. This was done separately for each participant and performed in functional space.

### Brain imaging maps

As control participants demonstrated slightly different hMT+ localization in left and right hemispheres, we decided to show hMT+ group maps for left and right V1 lesions separately. This generated a group size of *n* = 6 for blindsight-positive patients with left V1 lesions (Fig 1Ai), *n* = 5 for blindsight-negative patients with left V1 lesions (Fig 1Bi), *n* = 2 for blindsight positive-patients with right V1 lesions (Fig 1Aii), and *n* = 1 for blindsight-negative patients with a right V1 lesion (Fig 1Bii). Mixed effects analyses were used for all group analyses where *n* > 3. A statistical threshold of *p* < 0.001 uncorrected was used to test for significance within V1 and extrastriate cortex, for which there were a priori hypotheses. Elsewhere, correction for multiple comparisons was made using a cluster threshold of *p* < 0.05 unless otherwise stated.

### Quantitative statistics

Statistical tests to quantify differences in functional activity and co-activation between ROIs or participant groups were implemented in Excel or MATLAB. For overall hMT+ percent BOLD responses (Fig 1, bar charts), activity was averaged across all five motion speeds (0–32°/s). Two statistical analyses were then performed: (i) mean activity comparing sighted and blind hemifield (and hemisphere) used a paired *t* test and (ii) mean activity compared to baseline using a one-sample *t* test versus zero. A two-way ANOVA was also used to assess the effect of participant group and speed on blind hemifield responses (right hemifield in controls) and separately on sighted hemifield responses (left hemifield in controls).

In fMRI time series correlations, it is known that task conditions can influence intrinsic temporal correlations (e.g., see [12,17]). To ensure that correlations only reflected resting block activity, we used the residuals timeseries for ROIs once stimulus responses had been regressed out. This allowed us to determine resting ROI1 versus ROI2 correlation analyses for each participant.

For correlation analyses, two main statistical methods were used. For correlations between participant groups, a Pearson correlation coefficient was derived from mean activity in ROI1 versus ROI2 at each level of speed, i.e., *n* = 5. For correlations between ROIs within participant groups, a Pearson correlation coefficient was determined separately for each participant (activity in ROI1 versus ROI2, at each level of speed). We then calculated weighted averages of r coefficients for the group, using a Fischer transformation to approximate correlations to a normally distributed measure (Figs 2 and 3). A significant effect of group was determined by performing a one-way ANOVA. Pairwise comparisons between participant groups or ROIs were then calculated using post-hoc *t* tests. One-sample *t* tests were used to compare r coefficients to zero. Whenever activity was compared in the same participant, a paired correlation analysis was performed.

### Seed region correlation maps

For each participant, the raw signal time series for the seed ROI was entered into the model as an explanatory variable. This was applied to the filtered and motion corrected whole brain timecourse. Stimulus conditions were also entered as regressors so that the model would better describe the data. Since the model was identical to the ROI time series, the parameter estimate was always 1 in the seed region. Any participant with voxels outside the seed region with a parameter estimate >1.5 were excluded from analysis, as these results were likely to be driven by noise. This was only a problem when using subcortical structures as seeds, as these are small regions with relatively weak signal that are more susceptible to artefact. This led to the exclusion of three participants from subcortical seed analyses (both hemispheres); two were blindsight positive (P5, P10), and one was blindsight negative (P7). Corroborating this, the raw LGN signal range in those three participants was significantly greater than the other 11 patients (66.6 ± SD 10.5 versus 39.6 ± SD 6.07, *t* = 2.2, *p* = 0.04) and controls (30.6 ± 1.9, *t* = 5.2, *p* < 0.001, df = 12).

To generate seed region correlation maps (Figs 4 and 5), the COPEs for each included participant were entered into a higher-level mixed effects analysis, and output parameter estimate (beta) maps were used to represent seed region correlation maps. This was performed separately for each participant group and for each seed region. The resulting maps were not intended to determine statistical significance but to allow visual inspection of the results from the separate groups. Visualisation thresholds were based upon control participants, and an optimal cutoff was used to display correlations in either V1, visual subcortex, and/or hMT+ without excess background noise. The same thresholds were applied to all participant groups. For LGN-oriented maps (Fig 5Aiv–5Avi and 5Biv–5Bvi), although not shown, no other subcortical regions showed equivalent beta levels, although ventral pulvinar was co-activated in both hemispheres of all participant groups when using a reduced threshold of 0.32.

## Supporting information

S1 Table Pathology location and patient demographics. Descriptions include pathology nature and anatomical location, gender, age at participation in the study, time since pathology onset (months), visual field deficit, and blindsight status. HH = homonymous hemianopia, L = left, M = months, R = right, UQ = upper quadrantanopia.
(DOCX)

S1 Fig Behavioural paradigm and results. **(A)** Experiment 1: 2AFC temporal detection. Patients fixated on a central cross, with onset of each 1,500-ms interval alerted by a low (interval 1) or high (interval 2) pitch tone. Stimuli were located inside the scotoma (see S6 Fig) and could appear in either interval at random for a period of 500 ms. At the end of the trial, participants had to decide in which interval it appeared. Stimuli consisted of an aperture of 5° or 8° diameter, containing static or moving black dots (speed 0, 4, 8, 20, or 32°/s, at random). **(B)** Experiment 2: 2AFC direction discrimination. Throughout each trial of 2,500-ms duration, participants were required to fixate on a central black cross. During this time, the stimulus appeared inside the scotoma for 500 ms with jittered onset. At the end of the trial, patients had to indicate which direction the dots were moving (horizontal or vertical). If they saw nothing, they were instructed to guess. Controls did not perform behavioural experiments, as they would be at ceiling. **(C)** Mean behavioural performance ± SEM for 2AFC temporal detection, as a function of stimulus speed. **(D)** Mean behavioural performance ± SEM for 2AFC direction discrimination, as a function of stimulus speed. Results for blindsight-positive patients (pink diamond) and blindsight-negative patients (blue circle) are shown separately. Dashed grey line represents chance level (50%), with statistical symbols representing group-level one-tailed *t* tests versus chance (*μ*: *p < 0*.*01*, *: *p* < 0.05). All other values were nonsignificant. Underlying data can be found in S4 Data. 2AFC, two-alternate forced choice; SEM, standard error of the mean.
(PNG)

S2 Fig fMRI procedure and group lesion maps. **(A)** Stimulus size and position for all patients and controls. Each transparent circle represents the stimulus aperture for a single participant. The black cross at coordinates (0,0) represents fixation. Only the right hemifield is shown, but stimuli were also presented to precisely equivalent locations in the opposite hemifield. **(B)** Simple block design, presenting an aperture of black dots to the blind portion of visual field or its equivalent location in the sighted hemifield. Stimuli had identical parameters to behavioural testing. Stimulus speed in each block was randomized to one of five levels (0, 4, 8, 20, and 32°/s). This represented 10 conditions in total, with each block lasting 16 s with 10-s rest periods. **(C)** Throughout all blocks, a fixation task required participants to press a button every time the central fixation cross changed colour from black to red. Colour changes occurred at random lasting 300 ms. All participants scored at least 90%, with mean performance and SEM plotted for patients and controls. **(D)** Summed lesion maps for blindsight-positive patients on standard-space MNI template brain. **(E)** Summed lesion maps for blindsight-negative patients, on standard space MNI template brain. Colour scale represents the number of patients with lesions involving that voxel, from 1 to 8 in blindsight-positive patients and 1 to 6 in blindsight-negative patients. Patients with right hemisphere lesions (*n* = 3) had structural scans flipped in the horizontal plane to allow images to be aligned and aid visualisation, using radiological convention. Shaded blue areas represent binarized Jülich-defined probabilistic maps of hMT+. Underlying data for panels A and C can be found in S5 Data. fMRI, functional MRI; MNI, Montreal Neurological Institute; SEM, standard error of the mean.
(TIF)

S3 Fig Subcortical ROIs in native (functional), structural, and standard space for all 14 patients. Slices in each ‘space’ are matched to show equivalent, representative views. Ventral pulvinar (yellow) and SC (blue) ROIs are depicted in the upper rows and LGN (green) in lower rows. Background brain images in structural space are the T1-weighted MPRAGE images. These have been transformed to native (functional) and standard space for background images, radiological convention. LHF refers to blind left hemifield, RHF refers to blind right hemifield, BS+ is blindsight positive, and BS- is blindsight negative. LGN, lateral geniculate nucleus; ROI, region of interest; SC, superior colliculus.
(PNG)

S4 Fig Individual lesion maps. **(A)** Blindsight-positive patients. **(B)** Blindsight-negative patients. Lesions are highlighted with red-yellow masks, and shaded blue areas represent binarized Jülich-defined probabilistic maps of hMT+. Background images are structural T1 scans, radiological convention.
(PNG)

S5 Fig Lesion involvement of hMT+ and its surrounding white matter. **(A)** Example of P6 and **(B)** P12, illustrating the isotropic 40 × 40 × 40mm^3^ cuboidal ROI (green), centred on the ipsilesional hMT+ mask (blue), which includes hMT+ surrounding white matter. Where the ROI overlaps with the lesion mask, voxels are coloured red (no overlap in panel A). Background brain images are structural T1 scans, radiological convention. **(C)** The voxel count for regions of overlap between the hMT+/white matter ROI and lesion masks for each patient. ROI, region of interest.
(PNG)

S6 Fig Visual field loss and T1 structural images. In each patient, perimetry reports are depicted schematically showing the location of target stimuli. Dense visual field loss is shown in black (<0.5%) and partial loss in grey (<2%). Stimuli were restricted to a region of dense visual field loss, a minimum of 2.5 degrees from fixation. Concentric rings represent increments in retinal position of 10 degrees, spanning the central 30 degrees. Equivalent perimetry data (Humphrey 30:2 except P10, who has Goldmann) are shown alongside (outer columns) where available. Blindsight status and estimates of the percentage of scotoma covered by the stimulus (percent) are provided for each patient. Representative T1 structural axial slices demonstrate the lesion location, using radiological convention. Of note, patients P3, P8, P10, P11, and P13 took part in a previous study [33], in which they demonstrated significant blindsight performance for detection of a drifting Gabor.
(PNG)

S1 Data Mean signal change (%) in contralateral hMT+, (1) averaged across all 5 speed conditions, (2) as a function of stimulus speed, for each hemifield in patients and controls. This data underlies Fig 1iii and 1iv.
(XLSX)

S2 Data Residual BOLD signal in the ROIs, for every scan volume. Each tab represents data for one participant. This data underlies Fig 2. BOLD, blood oxygen level–dependent; ROI, region of interest.
(XLSX)

S3 Data Residual BOLD signal in the ROIs, for every scan volume. Each tab represents data for one participant. This data underlies Fig 3. BOLD, blood oxygen level–dependent; ROI, region of interest.
(XLSX)

S4 Data Mean behavioural performance for 2AFC detection and discrimination tests, for each participant. This data underlies S1 Fig. 2AFC, two-alternate forced choice.
(XLSX)

S5 Data (1) Position and size of stimulus apertures, and (2) performance on the fMRI fixation task, for each participant. This data underlies S2A and S2C Fig. fMRI, functional MRI.
(XLSX)

## Abbreviations

2AFC: two-alternate forced choice
BET: Brain Extraction Tool
BOLD: blood oxygen level–dependent
COPE: contrast of parameter estimate
fMRI: functional MRI
FMRIB: Functional Magnetic Resonance Imaging Centre of the Brain
FSL: FMRIB's Software Library
LGN: lateral geniculate nucleus
MNI: Montreal Neurological Institute
MT: middle temporal area
ROI: region of interest
SC: superior colliculus
SEM: standard error of the mean
V1: primary visual cortex
VFI: Visual Field Index
