# Supplementary material for: Blindsight relies on a functional connection between hMT+ and the lateral geniculate nucleus, not the pulvinar
Source: PLoS Biol. 2018 Jul 25;16(7):e2005769. doi: 10.1371/journal.pbio.2005769 (PMC6078309; doi:10.1371/journal.pbio.2005769)
Supplement: S1 Table — Descriptions include pathology nature and anatomical location, gender, age at participation in the study, time since pathology onset (months), visual field deficit, and blindsight status. HH = homonymous hemianopia, L = left, M = months, R = right, UQ = upper quadrantanopia. (DOCX) [file pbio.2005769.s001.docx]

| **Patient** | **Age** | **Pathology** | **Time since lesion (M)** | **Visual field deficit** | **Blindsight status** | **Gender**  **(F/M)** |
| --- | --- | --- | --- | --- | --- | --- |
| **P1** | 76 | Left occipital tumour resection sparing lateral regions of the occipital lobe including extrastriate cortex | 252 | RHH | Negative | M |
| **P2** | 66 | Left occipital infarct involving lingual gyrus and small portion of white matter | 8 | RHH | Positive | M |
| **P3** | 67 | Right occipito-temporal haemorrhage mostly restricted to right lingual gyrus | 6 | LHH | Positive | F |
| **P4** | 45 | Left occipital infarct with damage restricted to grey matter in the medial portion of the left occipital lobe | 7 | RHH | Negative | F |
| **P5** | 68 | Right occipital infarct with damage mostly restricted to the right lingual gyrus, including a small portion of white matter | 16 | LHH | Positive | M |
| **P6** | 56 | Left occipital infarct involving medial occipital cortex and extension to the left cerebellum | 18 | RHH | Negative | M |
| **P7** | 48 | Large iatrogenic left occipital infarct encompassing medial and ventral occipital cortex, with extension into the temporal lobe but sparing dorsolateral regions | 84 | RHH | Negative | M |
| **P8** | 38 | Left occipital infarct with damage isolated to the medial aspect of the left occipital lobe | 7 | RHH | Positive | M |
| **P9** | 71 | Left occipital infarct involving cortex and white matter in the medial left temporal and occipital lobes | 19 | RUQ | Negative | M |
| **P10** | 60 | Left occipital infarct with damage restricted to the posterior-medial left occipital lobe | 96 | RHH | Positive | M |
| **P11** | 31 | Left occipital infarct with damage restricted to the medial portion of the left occipital lobe | 156 | RHH | Positive | M |
| **P12** | 72 | Right occipital haemorrhage involving extensive grey and white matter in the right occipital lobe and a small region of right parietal lobe medially | 6 | LHH | Negative | M |
| **P13** | 41 | Left occipital infarct involving medial and inferior portions of the left occipital lobe | 6 | RHH | Positive | F |
| **P14** | 38 | Left occipital infarct restricted to the medial aspect of the left occipital lobe | 7 | RHH | Positive | F |

**S1 Table. Pathology location and patient demographics.** Descriptions include pathology nature and anatomical location, gender, age at participation in the study, time since pathology onset (months), visual field deficit, and blindsight status. M = months, HH = homonymous hemianopia, L = left, R = right, UQ = upper quadrantanopia.
