# Supplementary figures and images for: Blindsight relies on a functional connection between hMT+ and the lateral geniculate nucleus, not the pulvinar
Source: PLoS Biol. 2018 Jul 25;16(7):e2005769. doi: 10.1371/journal.pbio.2005769 (PMC6078309; doi:10.1371/journal.pbio.2005769)

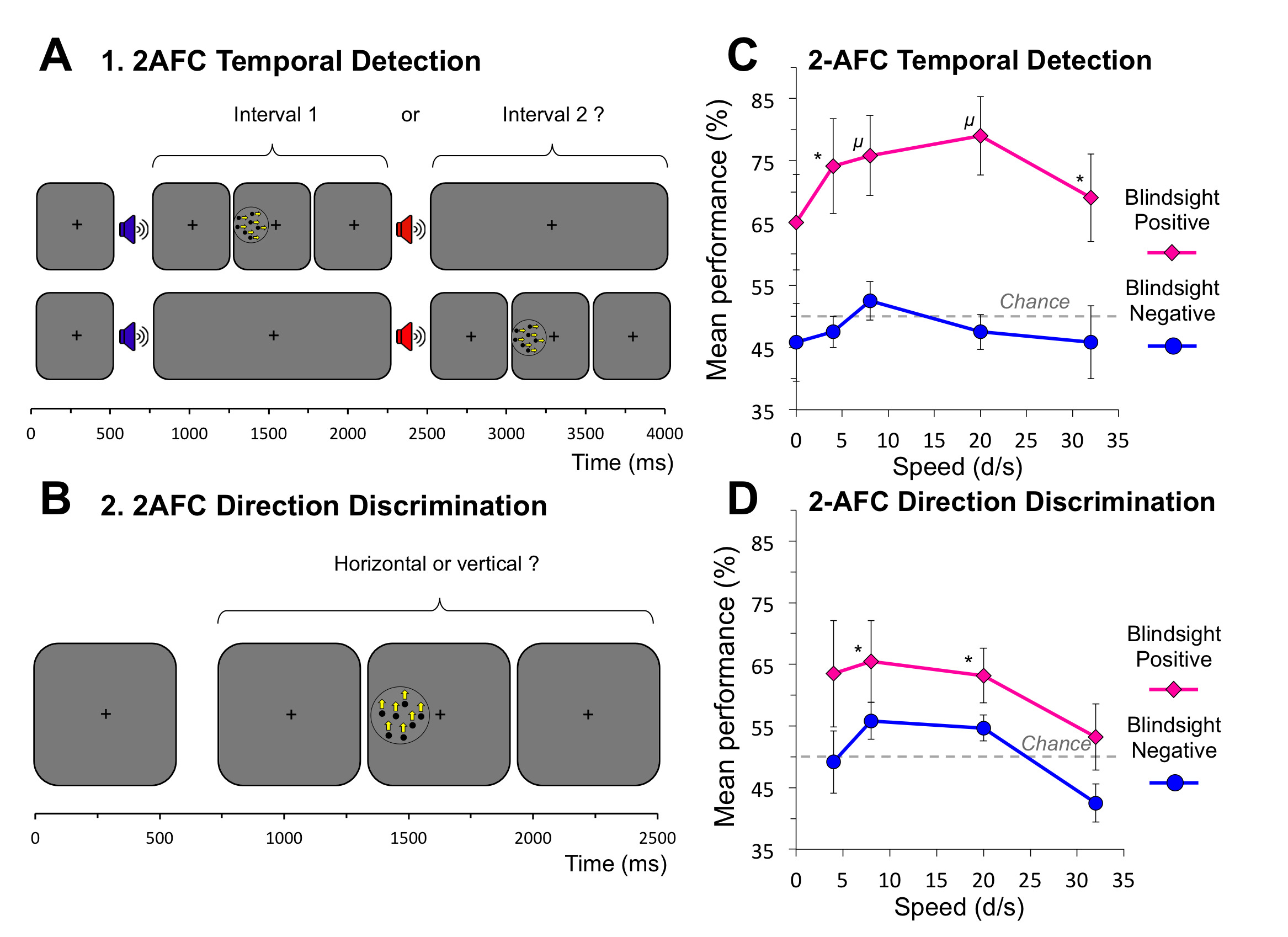

Supplement: S1 Fig — (A) Experiment 1: 2AFC temporal detection. Patients fixated on a central cross, with onset of each 1,500-ms interval alerted by a low (interval 1) or high (interval 2) pitch tone. Stimuli were located inside the scotoma (see S6 Fig) and could appear in either interval at random for a period of 500 ms. At the end of the trial, participants had to decide in which interval it appeared. Stimuli consisted of an aperture of 5° or 8° diameter, containing static or moving black dots (speed 0, 4, 8, 20, or 32°/s, at random). (B) Experiment 2: 2AFC direction discrimination. Throughout each trial of 2,500-ms duration, participants were required to fixate on a central black cross. During this time, the stimulus appeared inside the scotoma for 500 ms with jittered onset. At the end of the trial, patients had to indicate which direction the dots were moving (horizontal or vertical). If they saw nothing, they were instructed to guess. Controls did not perform behavioural experiments, as they would be at ceiling. (C) Mean behavioural performance ± SEM for 2AFC temporal detection, as a function of stimulus speed. (D) Mean behavioural performance ± SEM for 2AFC direction discrimination, as a function of stimulus speed. Results for blindsight-positive patients (pink diamond) and blindsight-negative patients (blue circle) are shown separately. Dashed grey line represents chance level (50%), with statistical symbols representing group-level one-tailed t tests versus chance (μ: p < 0.01, *: p < 0.05). All other values were nonsignificant. Underlying data can be found in S4 Data. 2AFC, two-alternate forced choice; SEM, standard error of the mean. (PNG) [file pbio.2005769.s002.png]

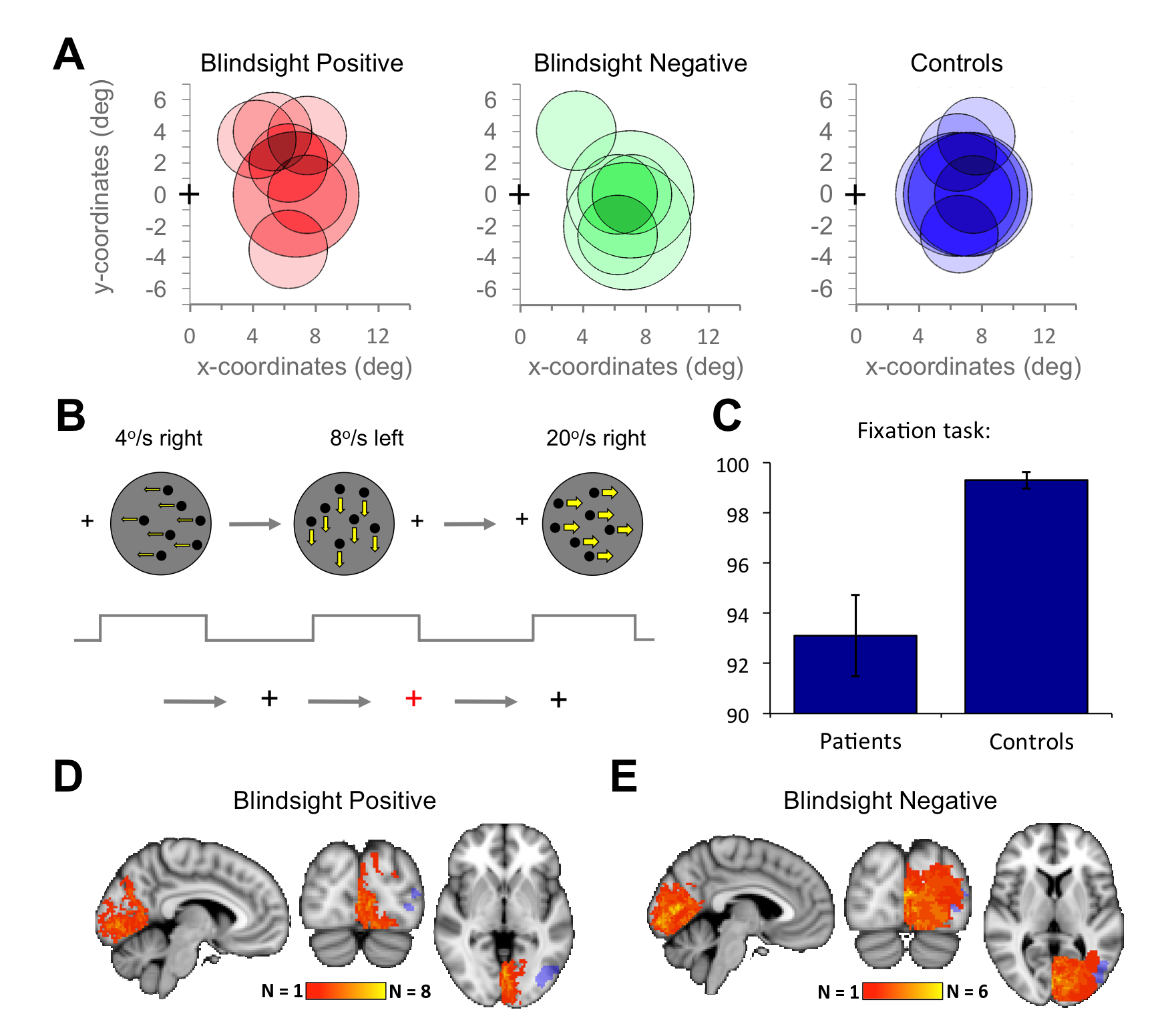

Supplement: S2 Fig — (A) Stimulus size and position for all patients and controls. Each transparent circle represents the stimulus aperture for a single participant. The black cross at coordinates (0,0) represents fixation. Only the right hemifield is shown, but stimuli were also presented to precisely equivalent locations in the opposite hemifield. (B) Simple block design, presenting an aperture of black dots to the blind portion of visual field or its equivalent location in the sighted hemifield. Stimuli had identical parameters to behavioural testing. Stimulus speed in each block was randomized to one of five levels (0, 4, 8, 20, and 32°/s). This represented 10 conditions in total, with each block lasting 16 s with 10-s rest periods. (C) Throughout all blocks, a fixation task required participants to press a button every time the central fixation cross changed colour from black to red. Colour changes occurred at random lasting 300 ms. All participants scored at least 90%, with mean performance and SEM plotted for patients and controls. (D) Summed lesion maps for blindsight-positive patients on standard-space MNI template brain. (E) Summed lesion maps for blindsight-negative patients, on standard space MNI template brain. Colour scale represents the number of patients with lesions involving that voxel, from 1 to 8 in blindsight-positive patients and 1 to 6 in blindsight-negative patients. Patients with right hemisphere lesions (n = 3) had structural scans flipped in the horizontal plane to allow images to be aligned and aid visualisation, using radiological convention. Shaded blue areas represent binarized Jülich-defined probabilistic maps of hMT+. Underlying data for panels A and C can be found in S5 Data. fMRI, functional MRI; MNI, Montreal Neurological Institute; SEM, standard error of the mean. (TIF) [file pbio.2005769.s003.tif]

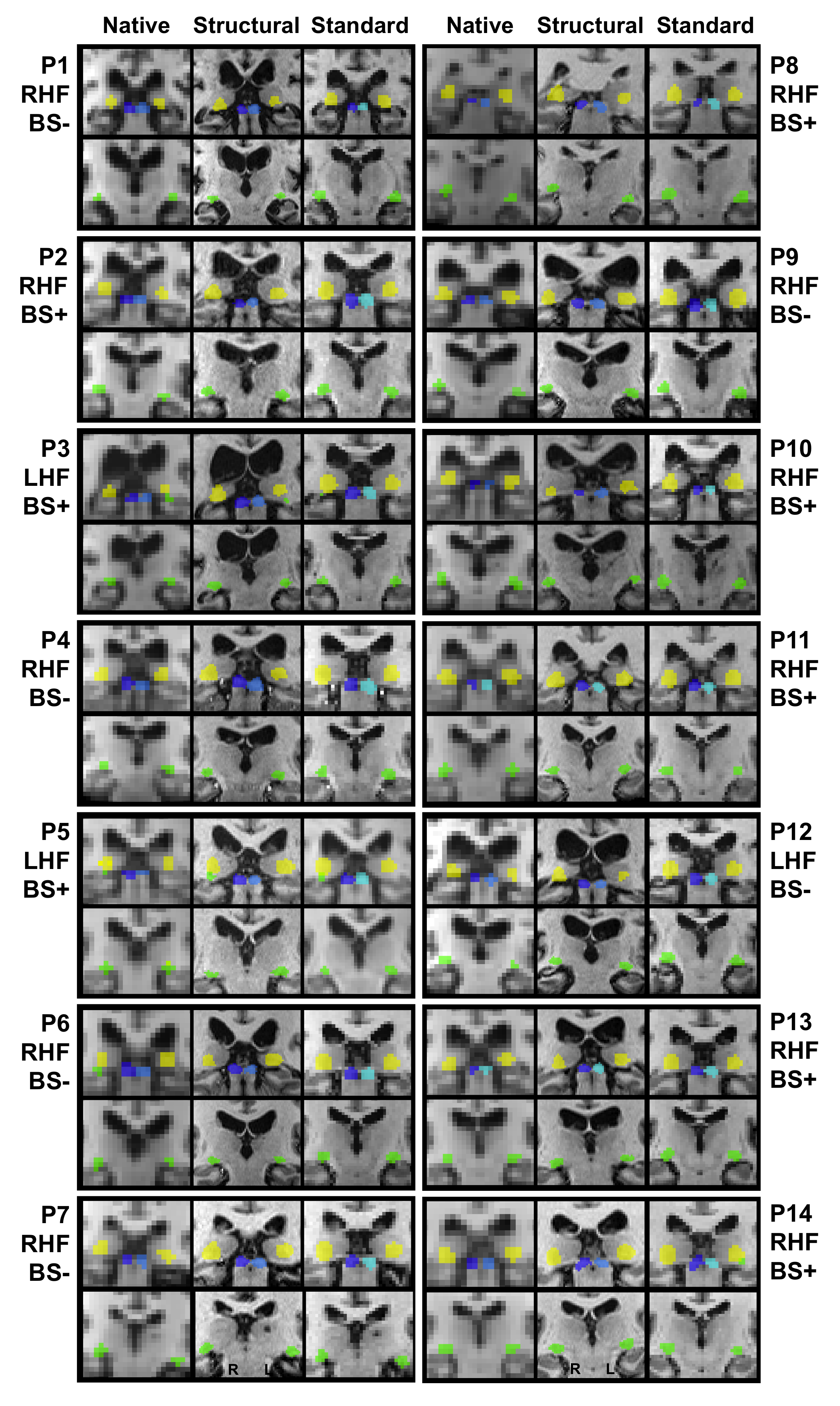

Supplement: S3 Fig — Slices in each ‘space’ are matched to show equivalent, representative views. Ventral pulvinar (yellow) and SC (blue) ROIs are depicted in the upper rows and LGN (green) in lower rows. Background brain images in structural space are the T1-weighted MPRAGE images. These have been transformed to native (functional) and standard space for background images, radiological convention. LHF refers to blind left hemifield, RHF refers to blind right hemifield, BS+ is blindsight positive, and BS- is blindsight negative. LGN, lateral geniculate nucleus; ROI, region of interest; SC, superior colliculus. (PNG) [file pbio.2005769.s004.png]

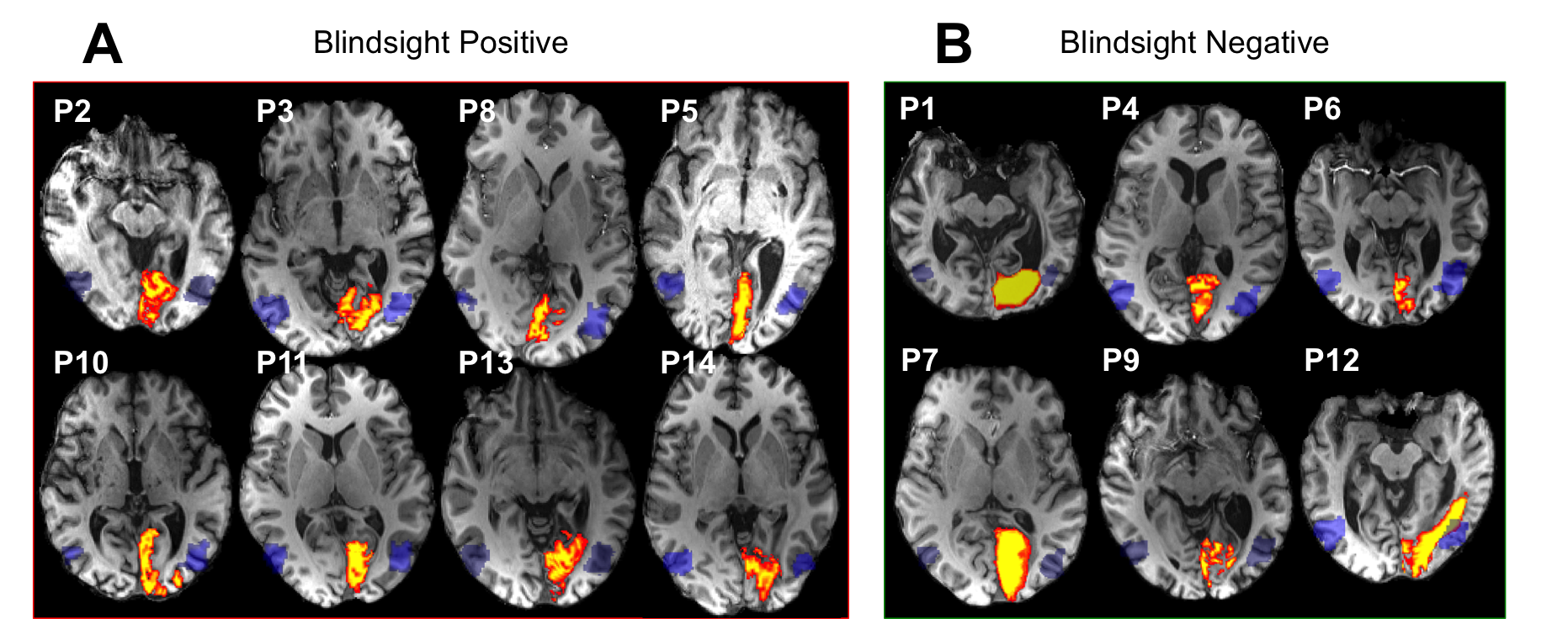

Supplement: S4 Fig — (A) Blindsight-positive patients. (B) Blindsight-negative patients. Lesions are highlighted with red-yellow masks, and shaded blue areas represent binarized Jülich-defined probabilistic maps of hMT+. Background images are structural T1 scans, radiological convention. (PNG) [file pbio.2005769.s005.png]

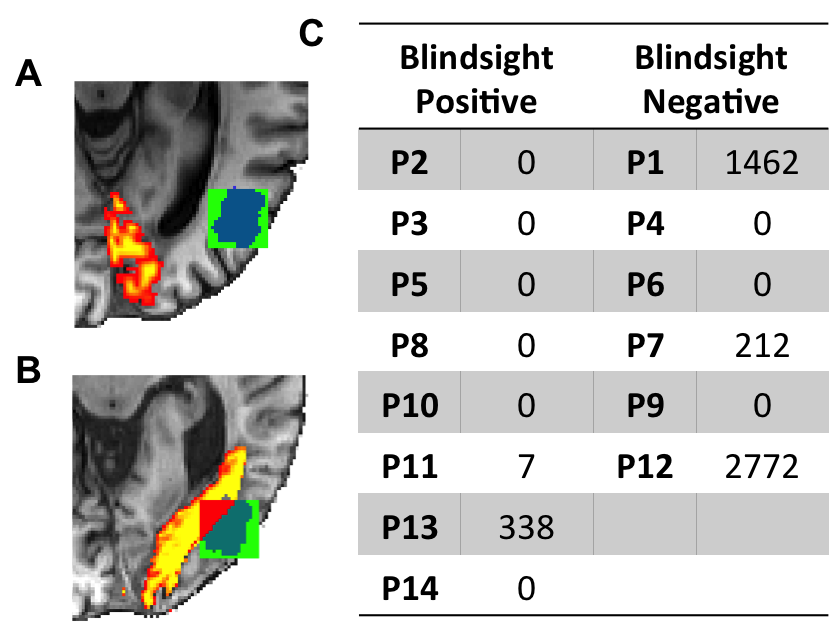

Supplement: S5 Fig — (A) Example of P6 and (B) P12, illustrating the isotropic 40 × 40 × 40mm3 cuboidal ROI (green), centred on the ipsilesional hMT+ mask (blue), which includes hMT+ surrounding white matter. Where the ROI overlaps with the lesion mask, voxels are coloured red (no overlap in panel A). Background brain images are structural T1 scans, radiological convention. (C) The voxel count for regions of overlap between the hMT+/white matter ROI and lesion masks for each patient. ROI, region of interest. (PNG) [file pbio.2005769.s006.png]

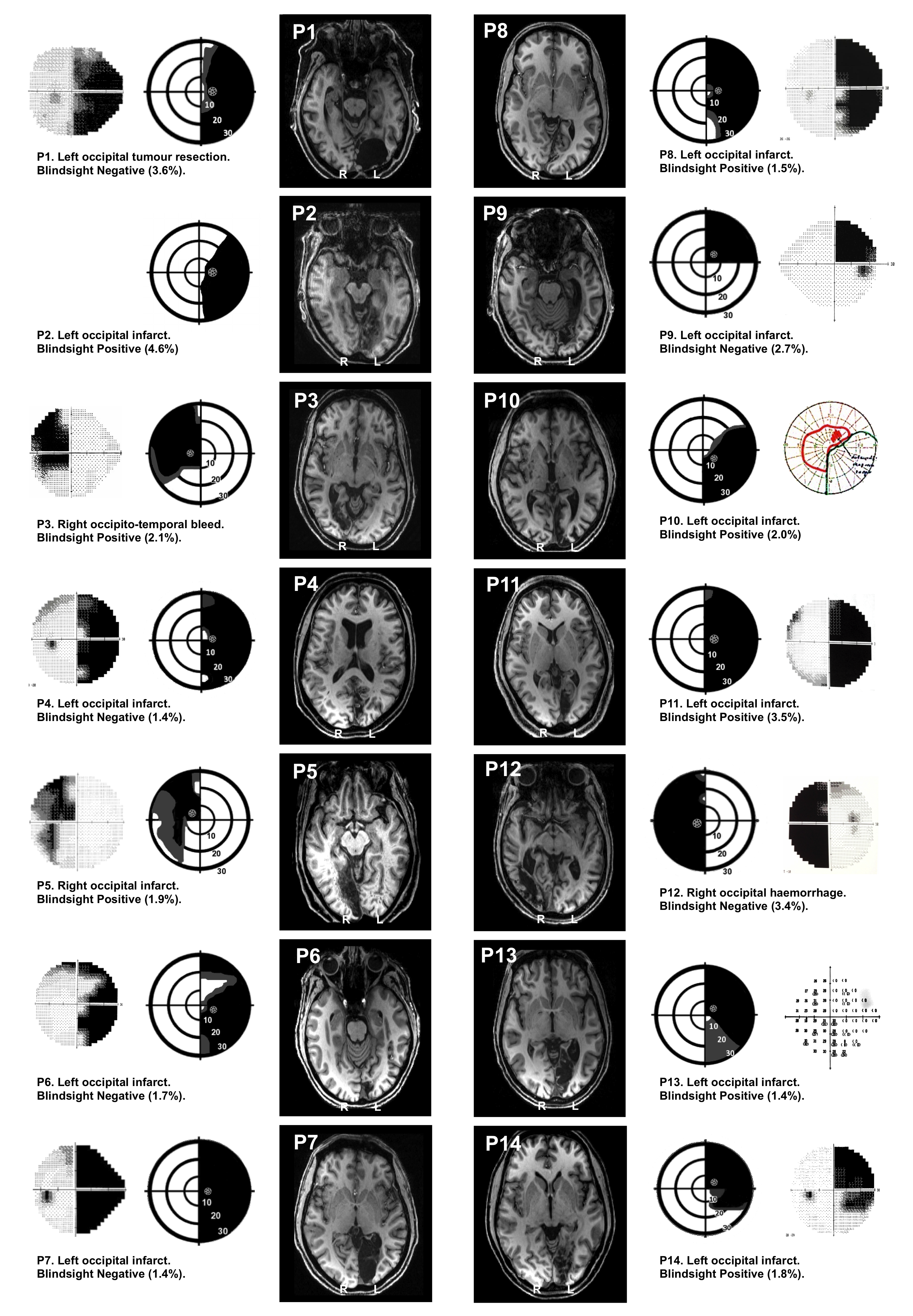

Supplement: S6 Fig — In each patient, perimetry reports are depicted schematically showing the location of target stimuli. Dense visual field loss is shown in black (<0.5%) and partial loss in grey (<2%). Stimuli were restricted to a region of dense visual field loss, a minimum of 2.5 degrees from fixation. Concentric rings represent increments in retinal position of 10 degrees, spanning the central 30 degrees. Equivalent perimetry data (Humphrey 30:2 except P10, who has Goldmann) are shown alongside (outer columns) where available. Blindsight status and estimates of the percentage of scotoma covered by the stimulus (percent) are provided for each patient. Representative T1 structural axial slices demonstrate the lesion location, using radiological convention. Of note, patients P3, P8, P10, P11, and P13 took part in a previous study [33], in which they demonstrated significant blindsight performance for detection of a drifting Gabor. (PNG) [file pbio.2005769.s007.png]
